# Measurement of the azimuthal anisotropy of charged particles produced in $\sqrt{s_{_\text {NN}}}$ = 5.02 TeV Pb+Pb collisions with the ATLAS detector

**DOI:** 10.1140/epjc/s10052-018-6468-7

**Published:** 2018-12-08

**Authors:** M. Aaboud, G. Aad, B. Abbott, O. Abdinov, B. Abeloos, D. K. Abhayasinghe, S. H. Abidi, O. S. AbouZeid, N. L. Abraham, H. Abramowicz, H. Abreu, Y. Abulaiti, B. S. Acharya, S. Adachi, L. Adamczyk, J. Adelman, M. Adersberger, A. Adiguzel, T. Adye, A. A. Affolder, Y. Afik, C. Agheorghiesei, J. A. Aguilar-Saavedra, F. Ahmadov, G. Aielli, S. Akatsuka, T. P. A. Åkesson, E. Akilli, A. V. Akimov, G. L. Alberghi, J. Albert, P. Albicocco, M. J. Alconada Verzini, S. Alderweireldt, M. Aleksa, I. N. Aleksandrov, C. Alexa, T. Alexopoulos, M. Alhroob, B. Ali, G. Alimonti, J. Alison, S. P. Alkire, C. Allaire, B. M. M. Allbrooke, B. W. Allen, P. P. Allport, A. Aloisio, A. Alonso, F. Alonso, C. Alpigiani, A. A. Alshehri, M. I. Alstaty, B. Alvarez Gonzalez, D. Álvarez Piqueras, M. G. Alviggi, B. T. Amadio, Y. Amaral Coutinho, L. Ambroz, C. Amelung, D. Amidei, S. P. Amor Dos Santos, S. Amoroso, C. S. Amrouche, C. Anastopoulos, L. S. Ancu, N. Andari, T. Andeen, C. F. Anders, J. K. Anders, K. J. Anderson, A. Andreazza, V. Andrei, C. R. Anelli, S. Angelidakis, I. Angelozzi, A. Angerami, A. V. Anisenkov, A. Annovi, C. Antel, M. T. Anthony, M. Antonelli, D. J. A. Antrim, F. Anulli, M. Aoki, J. A. Aparisi Pozo, L. Aperio Bella, G. Arabidze, J. P. Araque, V. Araujo Ferraz, R. Araujo Pereira, A. T. H. Arce, R. E. Ardell, F. A. Arduh, J.-F. Arguin, S. Argyropoulos, A. J. Armbruster, L. J. Armitage, A. Armstrong, O. Arnaez, H. Arnold, M. Arratia, O. Arslan, A. Artamonov, G. Artoni, S. Artz, S. Asai, N. Asbah, A. Ashkenazi, E. M. Asimakopoulou, L. Asquith, K. Assamagan, R. Astalos, R. J. Atkin, M. Atkinson, N. B. Atlay, K. Augsten, G. Avolio, R. Avramidou, M. K. Ayoub, G. Azuelos, A. E. Baas, M. J. Baca, H. Bachacou, K. Bachas, M. Backes, P. Bagnaia, M. Bahmani, H. Bahrasemani, A. J. Bailey, J. T. Baines, M. Bajic, C. Bakalis, O. K. Baker, P. J. Bakker, D. Bakshi Gupta, E. M. Baldin, P. Balek, F. Balli, W. K. Balunas, J. Balz, E. Banas, A. Bandyopadhyay, S. Banerjee, A. A. E. Bannoura, L. Barak, W. M. Barbe, E. L. Barberio, D. Barberis, M. Barbero, T. Barillari, M.-S. Barisits, J. Barkeloo, T. Barklow, N. Barlow, R. Barnea, S. L. Barnes, B. M. Barnett, R. M. Barnett, Z. Barnovska-Blenessy, A. Baroncelli, G. Barone, A. J. Barr, L. Barranco Navarro, F. Barreiro, J. Barreiro Guimarães da Costa, R. Bartoldus, A. E. Barton, P. Bartos, A. Basalaev, A. Bassalat, R. L. Bates, S. J. Batista, S. Batlamous, J. R. Batley, M. Battaglia, M. Bauce, F. Bauer, K. T. Bauer, H. S. Bawa, J. B. Beacham, T. Beau, P. H. Beauchemin, P. Bechtle, H. C. Beck, H. P. Beck, K. Becker, M. Becker, C. Becot, A. Beddall, A. J. Beddall, V. A. Bednyakov, M. Bedognetti, C. P. Bee, T. A. Beermann, M. Begalli, M. Begel, A. Behera, J. K. Behr, A. S. Bell, G. Bella, L. Bellagamba, A. Bellerive, M. Bellomo, P. Bellos, K. Belotskiy, N. L. Belyaev, O. Benary, D. Benchekroun, M. Bender, N. Benekos, Y. Benhammou, E. Benhar Noccioli, J. Benitez, D. P. Benjamin, M. Benoit, J. R. Bensinger, S. Bentvelsen, L. Beresford, M. Beretta, D. Berge, E. Bergeaas Kuutmann, N. Berger, L. J. Bergsten, J. Beringer, S. Berlendis, N. R. Bernard, G. Bernardi, C. Bernius, F. U. Bernlochner, T. Berry, P. Berta, C. Bertella, G. Bertoli, I. A. Bertram, G. J. Besjes, O. Bessidskaia Bylund, M. Bessner, N. Besson, A. Bethani, S. Bethke, A. Betti, A. J. Bevan, J. Beyer, R. M. Bianchi, O. Biebel, D. Biedermann, R. Bielski, K. Bierwagen, N. V. Biesuz, M. Biglietti, T. R. V. Billoud, M. Bindi, A. Bingul, C. Bini, S. Biondi, M. Birman, T. Bisanz, J. P. Biswal, C. Bittrich, D. M. Bjergaard, J. E. Black, K. M. Black, T. Blazek, I. Bloch, C. Blocker, A. Blue, U. Blumenschein, Dr. Blunier, G. J. Bobbink, V. S. Bobrovnikov, S. S. Bocchetta, A. Bocci, D. Boerner, D. Bogavac, A. G. Bogdanchikov, C. Bohm, V. Boisvert, P. Bokan, T. Bold, A. S. Boldyrev, A. E. Bolz, M. Bomben, M. Bona, J. S. Bonilla, M. Boonekamp, A. Borisov, G. Borissov, J. Bortfeldt, D. Bortoletto, V. Bortolotto, D. Boscherini, M. Bosman, J. D. Bossio Sola, K. Bouaouda, J. Boudreau, E. V. Bouhova-Thacker, D. Boumediene, C. Bourdarios, S. K. Boutle, A. Boveia, J. Boyd, D. Boye, I. R. Boyko, A. J. Bozson, J. Bracinik, N. Brahimi, A. Brandt, G. Brandt, O. Brandt, F. Braren, U. Bratzler, B. Brau, J. E. Brau, W. D. Breaden Madden, K. Brendlinger, A. J. Brennan, L. Brenner, R. Brenner, S. Bressler, B. Brickwedde, D. L. Briglin, D. Britton, D. Britzger, I. Brock, R. Brock, G. Brooijmans, T. Brooks, W. K. Brooks, E. Brost, J. H Broughton, P. A. Bruckman de Renstrom, D. Bruncko, A. Bruni, G. Bruni, L. S. Bruni, S. Bruno, B. H. Brunt, M. Bruschi, N. Bruscino, P. Bryant, L. Bryngemark, T. Buanes, Q. Buat, P. Buchholz, A. G. Buckley, I. A. Budagov, F. Buehrer, M. K. Bugge, O. Bulekov, D. Bullock, T. J. Burch, S. Burdin, C. D. Burgard, A. M. Burger, B. Burghgrave, K. Burka, S. Burke, I. Burmeister, J. T. P. Burr, D. Büscher, V. Büscher, E. Buschmann, P. Bussey, J. M. Butler, C. M. Buttar, J. M. Butterworth, P. Butti, W. Buttinger, A. Buzatu, A. R. Buzykaev, G. Cabras, S. Cabrera Urbán, D. Caforio, H. Cai, V. M. M. Cairo, O. Cakir, N. Calace, P. Calafiura, A. Calandri, G. Calderini, P. Calfayan, G. Callea, L. P. Caloba, S. Calvente Lopez, D. Calvet, S. Calvet, T. P. Calvet, M. Calvetti, R. Camacho Toro, S. Camarda, P. Camarri, D. Cameron, R. Caminal Armadans, C. Camincher, S. Campana, M. Campanelli, A. Camplani, A. Campoverde, V. Canale, M. Cano Bret, J. Cantero, T. Cao, Y. Cao, M. D. M. Capeans Garrido, I. Caprini, M. Caprini, M. Capua, R. M. Carbone, R. Cardarelli, F. C. Cardillo, I. Carli, T. Carli, G. Carlino, B. T. Carlson, L. Carminati, R. M. D. Carney, S. Caron, E. Carquin, S. Carrá, G. D. Carrillo-Montoya, D. Casadei, M. P. Casado, A. F. Casha, D. W. Casper, R. Castelijn, F. L. Castillo, V. Castillo Gimenez, N. F. Castro, A. Catinaccio, J. R. Catmore, A. Cattai, J. Caudron, V. Cavaliere, E. Cavallaro, D. Cavalli, M. Cavalli-Sforza, V. Cavasinni, E. Celebi, F. Ceradini, L. Cerda Alberich, A. S. Cerqueira, A. Cerri, L. Cerrito, F. Cerutti, A. Cervelli, S. A. Cetin, A. Chafaq, D. Chakraborty, S. K. Chan, W. S. Chan, Y. L. Chan, J. D. Chapman, B. Chargeishvili, D. G. Charlton, C. C. Chau, C. A. Chavez Barajas, S. Che, A. Chegwidden, S. Chekanov, S. V. Chekulaev, G. A. Chelkov, M. A. Chelstowska, C. Chen, C. H. Chen, H. Chen, J. Chen, J. Chen, S. Chen, S. J. Chen, X. Chen, Y. Chen, Y.-H. Chen, H. C. Cheng, H. J. Cheng, A. Cheplakov, E. Cheremushkina, R. Cherkaoui El Moursli, E. Cheu, K. Cheung, L. Chevalier, V. Chiarella, G. Chiarelli, G. Chiodini, A. S. Chisholm, A. Chitan, I. Chiu, Y. H. Chiu, M. V. Chizhov, K. Choi, A. R. Chomont, S. Chouridou, Y. S. Chow, V. Christodoulou, M. C. Chu, J. Chudoba, A. J. Chuinard, J. J. Chwastowski, L. Chytka, D. Cinca, V. Cindro, I. A. Cioară, A. Ciocio, F. Cirotto, Z. H. Citron, M. Citterio, A. Clark, M. R. Clark, P. J. Clark, C. Clement, Y. Coadou, M. Cobal, A. Coccaro, J. Cochran, A. E. C. Coimbra, L. Colasurdo, B. Cole, A. P. Colijn, J. Collot, P. Conde Muiño, E. Coniavitis, S. H. Connell, I. A. Connelly, S. Constantinescu, F. Conventi, A. M. Cooper-Sarkar, F. Cormier, K. J. R. Cormier, M. Corradi, E. E. Corrigan, F. Corriveau, A. Cortes-Gonzalez, M. J. Costa, D. Costanzo, G. Cottin, G. Cowan, B. E. Cox, J. Crane, K. Cranmer, S. J. Crawley, R. A. Creager, G. Cree, S. Crépé-Renaudin, F. Crescioli, M. Cristinziani, V. Croft, G. Crosetti, A. Cueto, T. Cuhadar Donszelmann, A. R. Cukierman, J. Cúth, S. Czekierda, P. Czodrowski, M. J. Da Cunha Sargedas De Sousa, C. Da Via, W. Dabrowski, T. Dado, S. Dahbi, T. Dai, F. Dallaire, C. Dallapiccola, M. Dam, G. D’amen, J. Damp, J. R. Dandoy, M. F. Daneri, N. P. Dang, N. D. Dann, M. Danninger, V. Dao, G. Darbo, S. Darmora, O. Dartsi, A. Dattagupta, T. Daubney, S. D’Auria, W. Davey, C. David, T. Davidek, D. R. Davis, E. Dawe, I. Dawson, K. De, R. De Asmundis, A. De Benedetti, M. De Beurs, S. De Castro, S. De Cecco, N. De Groot, P. de Jong, H. De la Torre, F. De Lorenzi, A. De Maria, D. De Pedis, A. De Salvo, U. De Sanctis, A. De Santo, K. De Vasconcelos Corga, J. B. De Vivie De Regie, C. Debenedetti, D. V. Dedovich, N. Dehghanian, M. Del Gaudio, J. Del Peso, Y. Delabat Diaz, D. Delgove, F. Deliot, C. M. Delitzsch, M. Della Pietra, D. Della Volpe, A. Dell’Acqua, L. Dell’Asta, M. Delmastro, C. Delporte, P. A. Delsart, D. A. DeMarco, S. Demers, M. Demichev, S. P. Denisov, D. Denysiuk, L. D’Eramo, D. Derendarz, J. E. Derkaoui, F. Derue, P. Dervan, K. Desch, C. Deterre, K. Dette, M. R. Devesa, P. O. Deviveiros, A. Dewhurst, S. Dhaliwal, F. A. Di Bello, A. Di Ciaccio, L. Di Ciaccio, W. K. Di Clemente, C. Di Donato, A. Di Girolamo, B. Di Micco, R. Di Nardo, K. F. Di Petrillo, R. Di Sipio, D. Di Valentino, C. Diaconu, M. Diamond, F. A. Dias, T. Dias Do Vale, M. A. Diaz, J. Dickinson, E. B. Diehl, J. Dietrich, S. Díez Cornell, A. Dimitrievska, J. Dingfelder, F. Dittus, F. Djama, T. Djobava, J. I. Djuvsland, M. A. B. Do Vale, M. Dobre, D. Dodsworth, C. Doglioni, J. Dolejsi, Z. Dolezal, M. Donadelli, J. Donini, A. D’onofrio, M. D’Onofrio, J. Dopke, A. Doria, M. T. Dova, A. T. Doyle, E. Drechsler, E. Dreyer, T. Dreyer, Y. Du, J. Duarte-Campderros, F. Dubinin, M. Dubovsky, A. Dubreuil, E. Duchovni, G. Duckeck, A. Ducourthial, O. A. Ducu, D. Duda, A. Dudarev, A. C. Dudder, E. M. Duffield, L. Duflot, M. Dührssen, C. Dülsen, M. Dumancic, A. E. Dumitriu, A. K. Duncan, M. Dunford, A. Duperrin, H. Duran Yildiz, M. Düren, A. Durglishvili, D. Duschinger, B. Dutta, D. Duvnjak, M. Dyndal, S. Dysch, B. S. Dziedzic, C. Eckardt, K. M. Ecker, R. C. Edgar, T. Eifert, G. Eigen, K. Einsweiler, T. Ekelof, M. El Kacimi, R. El Kosseifi, V. Ellajosyula, M. Ellert, F. Ellinghaus, A. A. Elliot, N. Ellis, J. Elmsheuser, M. Elsing, D. Emeliyanov, Y. Enari, J. S. Ennis, M. B. Epland, J. Erdmann, A. Ereditato, S. Errede, M. Escalier, C. Escobar, O. Estrada Pastor, A. I. Etienvre, E. Etzion, H. Evans, A. Ezhilov, M. Ezzi, F. Fabbri, L. Fabbri, V. Fabiani, G. Facini, R. M. Faisca Rodrigues Pereira, R. M. Fakhrutdinov, S. Falciano, P. J. Falke, S. Falke, J. Faltova, Y. Fang, M. Fanti, A. Farbin, A. Farilla, E. M. Farina, T. Farooque, S. Farrell, S. M. Farrington, P. Farthouat, F. Fassi, P. Fassnacht, D. Fassouliotis, M. Faucci Giannelli, A. Favareto, W. J. Fawcett, L. Fayard, O. L. Fedin, W. Fedorko, M. Feickert, S. Feigl, L. Feligioni, C. Feng, E. J. Feng, M. Feng, M. J. Fenton, A. B. Fenyuk, L. Feremenga, J. Ferrando, A. Ferrari, P. Ferrari, R. Ferrari, D. E. Ferreira de Lima, A. Ferrer, D. Ferrere, C. Ferretti, F. Fiedler, A. Filipčič, F. Filthaut, K. D. Finelli, M. C. N. Fiolhais, L. Fiorini, C. Fischer, W. C. Fisher, N. Flaschel, I. Fleck, P. Fleischmann, R. R. M. Fletcher, T. Flick, B. M. Flierl, L. M. Flores, L. R. Flores Castillo, F. M. Follega, N. Fomin, G. T. Forcolin, A. Formica, F. A. Förster, A. C. Forti, A. G. Foster, D. Fournier, H. Fox, S. Fracchia, P. Francavilla, M. Franchini, S. Franchino, D. Francis, L. Franconi, M. Franklin, M. Frate, M. Fraternali, D. Freeborn, S. M. Fressard-Batraneanu, B. Freund, W. S. Freund, D. Froidevaux, J. A. Frost, C. Fukunaga, E. Fullana Torregrosa, T. Fusayasu, J. Fuster, O. Gabizon, A. Gabrielli, A. Gabrielli, G. P. Gach, S. Gadatsch, P. Gadow, G. Gagliardi, L. G. Gagnon, C. Galea, B. Galhardo, E. J. Gallas, B. J. Gallop, P. Gallus, G. Galster, R. Gamboa Goni, K. K. Gan, S. Ganguly, J. Gao, Y. Gao, Y. S. Gao, C. García, J. E. García Navarro, J. A. García Pascual, M. Garcia-Sciveres, R. W. Gardner, N. Garelli, V. Garonne, K. Gasnikova, A. Gaudiello, G. Gaudio, I. L. Gavrilenko, A. Gavrilyuk, C. Gay, G. Gaycken, E. N. Gazis, C. N. P. Gee, J. Geisen, M. Geisen, M. P. Geisler, K. Gellerstedt, C. Gemme, M. H. Genest, C. Geng, S. Gentile, S. George, D. Gerbaudo, G. Gessner, S. Ghasemi, M. Ghasemi Bostanabad, M. Ghneimat, B. Giacobbe, S. Giagu, N. Giangiacomi, P. Giannetti, A. Giannini, S. M. Gibson, M. Gignac, D. Gillberg, G. Gilles, D. M. Gingrich, M. P. Giordani, F. M. Giorgi, P. F. Giraud, P. Giromini, G. Giugliarelli, D. Giugni, F. Giuli, M. Giulini, S. Gkaitatzis, I. Gkialas, E. L. Gkougkousis, P. Gkountoumis, L. K. Gladilin, C. Glasman, J. Glatzer, P. C. F. Glaysher, A. Glazov, M. Goblirsch-Kolb, J. Godlewski, S. Goldfarb, T. Golling, D. Golubkov, A. Gomes, R. Goncalves Gama, R. Gonçalo, G. Gonella, L. Gonella, A. Gongadze, F. Gonnella, J. L. Gonski, S. González de la Hoz, S. Gonzalez-Sevilla, L. Goossens, P. A. Gorbounov, H. A. Gordon, B. Gorini, E. Gorini, A. Gorišek, A. T. Goshaw, C. Gössling, M. I. Gostkin, C. A. Gottardo, C. R. Goudet, D. Goujdami, A. G. Goussiou, N. Govender, C. Goy, E. Gozani, I. Grabowska-Bold, P. O. J. Gradin, E. C. Graham, J. Gramling, E. Gramstad, S. Grancagnolo, V. Gratchev, P. M. Gravila, F. G. Gravili, C. Gray, H. M. Gray, Z. D. Greenwood, C. Grefe, K. Gregersen, I. M. Gregor, P. Grenier, K. Grevtsov, J. Griffiths, A. A. Grillo, K. Grimm, S. Grinstein, Ph. Gris, J.-F. Grivaz, S. Groh, E. Gross, J. Grosse-Knetter, G. C. Grossi, Z. J. Grout, C. Grud, A. Grummer, L. Guan, W. Guan, J. Guenther, A. Guerguichon, F. Guescini, D. Guest, R. Gugel, B. Gui, T. Guillemin, S. Guindon, U. Gul, C. Gumpert, J. Guo, W. Guo, Y. Guo, Z. Guo, R. Gupta, S. Gurbuz, G. Gustavino, B. J. Gutelman, P. Gutierrez, C. Gutschow, C. Guyot, M. P. Guzik, C. Gwenlan, C. B. Gwilliam, A. Haas, C. Haber, H. K. Hadavand, N. Haddad, A. Hadef, S. Hageböck, M. Hagihara, H. Hakobyan, M. Haleem, J. Haley, G. Halladjian, G. D. Hallewell, K. Hamacher, P. Hamal, K. Hamano, A. Hamilton, G. N. Hamity, K. Han, L. Han, S. Han, K. Hanagaki, M. Hance, D. M. Handl, B. Haney, R. Hankache, P. Hanke, E. Hansen, J. B. Hansen, J. D. Hansen, M. C. Hansen, P. H. Hansen, K. Hara, A. S. Hard, T. Harenberg, S. Harkusha, P. F. Harrison, N. M. Hartmann, Y. Hasegawa, A. Hasib, S. Hassani, S. Haug, R. Hauser, L. Hauswald, L. B. Havener, M. Havranek, C. M. Hawkes, R. J. Hawkings, D. Hayden, C. Hayes, C. P. Hays, J. M. Hays, H. S. Hayward, S. J. Haywood, M. P. Heath, V. Hedberg, L. Heelan, S. Heer, K. K. Heidegger, J. Heilman, S. Heim, T. Heim, B. Heinemann, J. J. Heinrich, L. Heinrich, C. Heinz, J. Hejbal, L. Helary, A. Held, S. Hellesund, S. Hellman, C. Helsens, R. C. W. Henderson, Y. Heng, S. Henkelmann, A. M. Henriques Correia, G. H. Herbert, H. Herde, V. Herget, Y. Hernández Jiménez, H. Herr, M. G. Herrmann, G. Herten, R. Hertenberger, L. Hervas, T. C. Herwig, G. G. Hesketh, N. P. Hessey, J. W. Hetherly, S. Higashino, E. Higón-Rodriguez, K. Hildebrand, E. Hill, J. C. Hill, K. K. Hill, K. H. Hiller, S. J. Hillier, M. Hils, I. Hinchliffe, M. Hirose, D. Hirschbuehl, B. Hiti, O. Hladik, D. R. Hlaluku, X. Hoad, J. Hobbs, N. Hod, M. C. Hodgkinson, A. Hoecker, M. R. Hoeferkamp, F. Hoenig, D. Hohn, D. Hohov, T. R. Holmes, M. Holzbock, M. Homann, S. Honda, T. Honda, T. M. Hong, A. Hönle, B. H. Hooberman, W. H. Hopkins, Y. Horii, P. Horn, A. J. Horton, L. A. Horyn, J.-Y. Hostachy, A. Hostiuc, S. Hou, A. Hoummada, J. Howarth, J. Hoya, M. Hrabovsky, J. Hrdinka, I. Hristova, J. Hrivnac, A. Hrynevich, T. Hryn’ova, P. J. Hsu, S.-C. Hsu, Q. Hu, S. Hu, Y. Huang, Z. Hubacek, F. Hubaut, M. Huebner, F. Huegging, T. B. Huffman, E. W. Hughes, M. Huhtinen, R. F. H. Hunter, P. Huo, A. M. Hupe, N. Huseynov, J. Huston, J. Huth, R. Hyneman, G. Iacobucci, G. Iakovidis, I. Ibragimov, L. Iconomidou-Fayard, Z. Idrissi, P. Iengo, R. Ignazzi, O. Igonkina, R. Iguchi, T. Iizawa, Y. Ikegami, M. Ikeno, D. Iliadis, N. Ilic, F. Iltzsche, G. Introzzi, M. Iodice, K. Iordanidou, V. Ippolito, M. F. Isacson, N. Ishijima, M. Ishino, M. Ishitsuka, W. Islam, C. Issever, S. Istin, F. Ito, J. M. Iturbe Ponce, R. Iuppa, A. Ivina, H. Iwasaki, J. M. Izen, V. Izzo, P. Jacka, P. Jackson, R. M. Jacobs, V. Jain, G. Jäkel, K. B. Jakobi, K. Jakobs, S. Jakobsen, T. Jakoubek, D. O. Jamin, D. K. Jana, R. Jansky, J. Janssen, M. Janus, P. A. Janus, G. Jarlskog, N. Javadov, T. Javůrek, M. Javurkova, F. Jeanneau, L. Jeanty, J. Jejelava, A. Jelinskas, P. Jenni, J. Jeong, S. Jézéquel, H. Ji, J. Jia, H. Jiang, Y. Jiang, Z. Jiang, S. Jiggins, F. A. Jimenez Morales, J. Jimenez Pena, S. Jin, A. Jinaru, O. Jinnouchi, H. Jivan, P. Johansson, K. A. Johns, C. A. Johnson, W. J. Johnson, K. Jon-And, R. W. L. Jones, S. D. Jones, S. Jones, T. J. Jones, J. Jongmanns, P. M. Jorge, J. Jovicevic, X. Ju, J. J. Junggeburth, A. Juste Rozas, A. Kaczmarska, M. Kado, H. Kagan, M. Kagan, T. Kaji, E. Kajomovitz, C. W. Kalderon, A. Kaluza, S. Kama, A. Kamenshchikov, L. Kanjir, Y. Kano, V. A. Kantserov, J. Kanzaki, B. Kaplan, L. S. Kaplan, D. Kar, M. J. Kareem, E. Karentzos, S. N. Karpov, Z. M. Karpova, V. Kartvelishvili, A. N. Karyukhin, L. Kashif, R. D. Kass, A. Kastanas, Y. Kataoka, C. Kato, J. Katzy, K. Kawade, K. Kawagoe, T. Kawamoto, G. Kawamura, E. F. Kay, V. F. Kazanin, R. Keeler, R. Kehoe, J. S. Keller, E. Kellermann, J. J. Kempster, J. Kendrick, O. Kepka, S. Kersten, B. P. Kerševan, R. A. Keyes, M. Khader, F. Khalil-Zada, A. Khanov, A. G. Kharlamov, T. Kharlamova, A. Khodinov, T. J. Khoo, E. Khramov, J. Khubua, S. Kido, M. Kiehn, C. R. Kilby, Y. K. Kim, N. Kimura, O. M. Kind, B. T. King, D. Kirchmeier, J. Kirk, A. E. Kiryunin, T. Kishimoto, D. Kisielewska, V. Kitali, O. Kivernyk, E. Kladiva, T. Klapdor-Kleingrothaus, M. H. Klein, M. Klein, U. Klein, K. Kleinknecht, P. Klimek, A. Klimentov, R. Klingenberg, T. Klingl, T. Klioutchnikova, F. F. Klitzner, P. Kluit, S. Kluth, E. Kneringer, E. B. F. G. Knoops, A. Knue, A. Kobayashi, D. Kobayashi, T. Kobayashi, M. Kobel, M. Kocian, P. Kodys, T. Koffas, E. Koffeman, N. M. Köhler, T. Koi, M. Kolb, I. Koletsou, T. Kondo, N. Kondrashova, K. Köneke, A. C. König, T. Kono, R. Konoplich, V. Konstantinides, N. Konstantinidis, B. Konya, R. Kopeliansky, S. Koperny, K. Korcyl, K. Kordas, A. Korn, I. Korolkov, E. V. Korolkova, O. Kortner, S. Kortner, T. Kosek, V. V. Kostyukhin, A. Kotwal, A. Koulouris, A. Kourkoumeli-Charalampidi, C. Kourkoumelis, E. Kourlitis, V. Kouskoura, A. B. Kowalewska, R. Kowalewski, T. Z. Kowalski, C. Kozakai, W. Kozanecki, A. S. Kozhin, V. A. Kramarenko, G. Kramberger, D. Krasnopevtsev, M. W. Krasny, A. Krasznahorkay, D. Krauss, J. A. Kremer, J. Kretzschmar, P. Krieger, K. Krizka, K. Kroeninger, H. Kroha, J. Kroll, J. Kroll, J. Krstic, U. Kruchonak, H. Krüger, N. Krumnack, M. C. Kruse, T. Kubota, S. Kuday, J. T. Kuechler, S. Kuehn, A. Kugel, F. Kuger, T. Kuhl, V. Kukhtin, R. Kukla, Y. Kulchitsky, S. Kuleshov, Y. P. Kulinich, M. Kuna, T. Kunigo, A. Kupco, T. Kupfer, O. Kuprash, H. Kurashige, L. L. Kurchaninov, Y. A. Kurochkin, M. G. Kurth, E. S. Kuwertz, M. Kuze, J. Kvita, T. Kwan, A. La Rosa, J. L. La Rosa Navarro, L. La Rotonda, F. La Ruffa, C. Lacasta, F. Lacava, J. Lacey, D. P. J. Lack, H. Lacker, D. Lacour, E. Ladygin, R. Lafaye, B. Laforge, T. Lagouri, S. Lai, S. Lammers, W. Lampl, E. Lançon, U. Landgraf, M. P. J. Landon, M. C. Lanfermann, V. S. Lang, J. C. Lange, R. J. Langenberg, A. J. Lankford, F. Lanni, K. Lantzsch, A. Lanza, A. Lapertosa, S. Laplace, J. F. Laporte, T. Lari, F. Lasagni Manghi, M. Lassnig, T. S. Lau, A. Laudrain, M. Lavorgna, A. T. Law, P. Laycock, M. Lazzaroni, B. Le, O. Le Dortz, E. Le Guirriec, E. P. Le Quilleuc, M. LeBlanc, T. LeCompte, F. Ledroit-Guillon, C. A. Lee, G. R. Lee, L. Lee, S. C. Lee, B. Lefebvre, M. Lefebvre, F. Legger, C. Leggett, N. Lehmann, G. Lehmann Miotto, W. A. Leight, A. Leisos, M. A. L. Leite, R. Leitner, D. Lellouch, B. Lemmer, K. J. C. Leney, T. Lenz, B. Lenzi, R. Leone, S. Leone, C. Leonidopoulos, G. Lerner, C. Leroy, R. Les, A. A. J. Lesage, C. G. Lester, M. Levchenko, J. Levêque, D. Levin, L. J. Levinson, D. Lewis, B. Li, C.-Q. Li, H. Li, L. Li, Q. Li, Q. Y. Li, S. Li, X. Li, Y. Li, Z. Liang, B. Liberti, A. Liblong, K. Lie, S. Liem, A. Limosani, C. Y. Lin, K. Lin, T. H. Lin, R. A. Linck, J. H. Lindon, B. E. Lindquist, A. L. Lionti, E. Lipeles, A. Lipniacka, M. Lisovyi, T. M. Liss, A. Lister, A. M. Litke, J. D. Little, B. Liu, B. L Liu, H. B. Liu, H. Liu, J. B. Liu, J. K. K. Liu, K. Liu, M. Liu, P. Liu, Y. Liu, Y. L. Liu, Y. W. Liu, M. Livan, A. Lleres, J. Llorente Merino, S. L. Lloyd, C. Y. Lo, F. Lo Sterzo, E. M. Lobodzinska, P. Loch, A. Loesle, T. Lohse, K. Lohwasser, M. Lokajicek, B. A. Long, J. D. Long, R. E. Long, L. Longo, K. A. Looper, J. A. Lopez, I. Lopez Paz, A. Lopez Solis, J. Lorenz, N. Lorenzo Martinez, M. Losada, P. J. Lösel, X. Lou, X. Lou, A. Lounis, J. Love, P. A. Love, J. J. Lozano Bahilo, H. Lu, M. Lu, N. Lu, Y. J. Lu, H. J. Lubatti, C. Luci, A. Lucotte, C. Luedtke, F. Luehring, I. Luise, L. Luminari, B. Lund-Jensen, M. S. Lutz, P. M. Luzi, D. Lynn, R. Lysak, E. Lytken, F. Lyu, V. Lyubushkin, H. Ma, L. L. Ma, Y. Ma, G. Maccarrone, A. Macchiolo, C. M. Macdonald, J. Machado Miguens, D. Madaffari, R. Madar, W. F. Mader, A. Madsen, N. Madysa, J. Maeda, K. Maekawa, S. Maeland, T. Maeno, A. S. Maevskiy, V. Magerl, C. Maidantchik, T. Maier, A. Maio, O. Majersky, S. Majewski, Y. Makida, N. Makovec, B. Malaescu, Pa. Malecki, V. P. Maleev, F. Malek, U. Mallik, D. Malon, C. Malone, S. Maltezos, S. Malyukov, J. Mamuzic, G. Mancini, I. Mandić, J. Maneira, L. Manhaes de Andrade Filho, J. Manjarres Ramos, K. H. Mankinen, A. Mann, A. Manousos, B. Mansoulie, J. D. Mansour, M. Mantoani, S. Manzoni, G. Marceca, L. March, L. Marchese, G. Marchiori, M. Marcisovsky, C. A. Marin Tobon, M. Marjanovic, D. E. Marley, F. Marroquim, Z. Marshall, M. U. F Martensson, S. Marti-Garcia, C. B. Martin, T. A. Martin, V. J. Martin, B. Martin dit Latour, M. Martinez, V. I. Martinez Outschoorn, S. Martin-Haugh, V. S. Martoiu, A. C. Martyniuk, A. Marzin, L. Masetti, T. Mashimo, R. Mashinistov, J. Masik, A. L. Maslennikov, L. H. Mason, L. Massa, P. Massarotti, P. Mastrandrea, A. Mastroberardino, T. Masubuchi, P. Mättig, J. Maurer, B. Maček, S. J. Maxfield, D. A. Maximov, R. Mazini, I. Maznas, S. M. Mazza, N. C. Mc Fadden, G. Mc Goldrick, S. P. Mc Kee, A. McCarn, T. G. McCarthy, L. I. McClymont, E. F. McDonald, J. A. Mcfayden, G. Mchedlidze, M. A. McKay, K. D. McLean, S. J. McMahon, P. C. McNamara, C. J. McNicol, R. A. McPherson, J. E. Mdhluli, Z. A. Meadows, S. Meehan, T. M. Megy, S. Mehlhase, A. Mehta, T. Meideck, B. Meirose, D. Melini, B. R. Mellado Garcia, J. D. Mellenthin, M. Melo, F. Meloni, A. Melzer, S. B. Menary, E. D. Mendes Gouveia, L. Meng, X. T. Meng, A. Mengarelli, S. Menke, E. Meoni, S. Mergelmeyer, C. Merlassino, P. Mermod, L. Merola, C. Meroni, F. S. Merritt, A. Messina, J. Metcalfe, A. S. Mete, C. Meyer, J. Meyer, J.-P. Meyer, H. Meyer Zu Theenhausen, F. Miano, R. P. Middleton, L. Mijović, G. Mikenberg, M. Mikestikova, M. Mikuž, M. Milesi, A. Milic, D. A. Millar, D. W. Miller, A. Milov, D. A. Milstead, A. A. Minaenko, M. Miñano Moya, I. A. Minashvili, A. I. Mincer, B. Mindur, M. Mineev, Y. Minegishi, Y. Ming, L. M. Mir, A. Mirto, K. P. Mistry, T. Mitani, J. Mitrevski, V. A. Mitsou, A. Miucci, P. S. Miyagawa, A. Mizukami, J. U. Mjörnmark, T. Mkrtchyan, M. Mlynarikova, T. Moa, K. Mochizuki, P. Mogg, S. Mohapatra, S. Molander, R. Moles-Valls, M. C. Mondragon, K. Mönig, J. Monk, E. Monnier, A. Montalbano, J. Montejo Berlingen, F. Monticelli, S. Monzani, N. Morange, D. Moreno, M. Moreno Llácer, P. Morettini, M. Morgenstern, S. Morgenstern, D. Mori, M. Morii, M. Morinaga, V. Morisbak, A. K. Morley, G. Mornacchi, A. P. Morris, J. D. Morris, L. Morvaj, P. Moschovakos, M. Mosidze, H. J. Moss, J. Moss, K. Motohashi, R. Mount, E. Mountricha, E. J. W. Moyse, S. Muanza, F. Mueller, J. Mueller, R. S. P. Mueller, D. Muenstermann, G. A. Mullier, F. J. Munoz Sanchez, P. Murin, W. J. Murray, A. Murrone, M. Muškinja, C. Mwewa, A. G. Myagkov, J. Myers, M. Myska, B. P. Nachman, O. Nackenhorst, K. Nagai, K. Nagano, Y. Nagasaka, M. Nagel, E. Nagy, A. M. Nairz, Y. Nakahama, K. Nakamura, T. Nakamura, I. Nakano, H. Nanjo, F. Napolitano, R. F. Naranjo Garcia, R. Narayan, D. I. Narrias Villar, I. Naryshkin, T. Naumann, G. Navarro, R. Nayyar, H. A. Neal, P. Y. Nechaeva, T. J. Neep, A. Negri, M. Negrini, S. Nektarijevic, C. Nellist, M. E. Nelson, S. Nemecek, P. Nemethy, M. Nessi, M. S. Neubauer, M. Neumann, P. R. Newman, T. Y. Ng, Y. S. Ng, H. D. N. Nguyen, T. Nguyen Manh, E. Nibigira, R. B. Nickerson, R. Nicolaidou, J. Nielsen, N. Nikiforou, V. Nikolaenko, I. Nikolic-Audit, K. Nikolopoulos, P. Nilsson, Y. Ninomiya, A. Nisati, N. Nishu, R. Nisius, I. Nitsche, T. Nitta, T. Nobe, Y. Noguchi, M. Nomachi, I. Nomidis, M. A. Nomura, T. Nooney, M. Nordberg, N. Norjoharuddeen, T. Novak, O. Novgorodova, R. Novotny, L. Nozka, K. Ntekas, E. Nurse, F. Nuti, F. G. Oakham, H. Oberlack, T. Obermann, J. Ocariz, A. Ochi, I. Ochoa, J. P. Ochoa-Ricoux, K. O’Connor, S. Oda, S. Odaka, S. Oerdek, A. Oh, S. H. Oh, C. C. Ohm, H. Oide, M. L. Ojeda, H. Okawa, Y. Okazaki, Y. Okumura, T. Okuyama, A. Olariu, L. F. Oleiro Seabra, S. A. Olivares Pino, D. Oliveira Damazio, J. L. Oliver, M. J. R. Olsson, A. Olszewski, J. Olszowska, D. C. O’Neil, A. Onofre, K. Onogi, P. U. E. Onyisi, H. Oppen, M. J. Oreglia, Y. Oren, D. Orestano, E. C. Orgill, N. Orlando, A. A. O’Rourke, R. S. Orr, B. Osculati, V. O’Shea, R. Ospanov, G. Otero y Garzon, H. Otono, M. Ouchrif, F. Ould-Saada, A. Ouraou, Q. Ouyang, M. Owen, R. E. Owen, V. E. Ozcan, N. Ozturk, J. Pacalt, H. A. Pacey, K. Pachal, A. Pacheco Pages, L. Pacheco Rodriguez, C. Padilla Aranda, S. Pagan Griso, M. Paganini, G. Palacino, S. Palazzo, S. Palestini, M. Palka, D. Pallin, I. Panagoulias, C. E. Pandini, J. G. Panduro Vazquez, P. Pani, G. Panizzo, L. Paolozzi, T. D. Papadopoulou, K. Papageorgiou, A. Paramonov, D. Paredes Hernandez, S. R. Paredes Saenz, B. Parida, A. J. Parker, K. A. Parker, M. A. Parker, F. Parodi, J. A. Parsons, U. Parzefall, V. R. Pascuzzi, J. M. P. Pasner, E. Pasqualucci, S. Passaggio, F. Pastore, P. Pasuwan, S. Pataraia, J. R. Pater, A. Pathak, T. Pauly, B. Pearson, M. Pedersen, L. Pedraza Diaz, R. Pedro, S. V. Peleganchuk, O. Penc, C. Peng, H. Peng, B. S. Peralva, M. M. Perego, A. P. Pereira Peixoto, D. V. Perepelitsa, F. Peri, L. Perini, H. Pernegger, S. Perrella, V. D. Peshekhonov, K. Peters, R. F. Y. Peters, B. A. Petersen, T. C. Petersen, E. Petit, A. Petridis, C. Petridou, P. Petroff, M. Petrov, F. Petrucci, M. Pettee, N. E. Pettersson, A. Peyaud, R. Pezoa, T. Pham, F. H. Phillips, P. W. Phillips, G. Piacquadio, E. Pianori, A. Picazio, M. A. Pickering, R. H. Pickles, R. Piegaia, J. E. Pilcher, A. D. Pilkington, M. Pinamonti, J. L. Pinfold, M. Pitt, M.-A. Pleier, V. Pleskot, E. Plotnikova, D. Pluth, P. Podberezko, R. Poettgen, R. Poggi, L. Poggioli, I. Pogrebnyak, D. Pohl, I. Pokharel, G. Polesello, A. Poley, A. Policicchio, R. Polifka, A. Polini, C. S. Pollard, V. Polychronakos, D. Ponomarenko, L. Pontecorvo, G. A. Popeneciu, D. M. Portillo Quintero, S. Pospisil, K. Potamianos, I. N. Potrap, C. J. Potter, H. Potti, T. Poulsen, J. Poveda, T. D. Powell, M. E. Pozo Astigarraga, P. Pralavorio, S. Prell, D. Price, M. Primavera, S. Prince, N. Proklova, K. Prokofiev, F. Prokoshin, S. Protopopescu, J. Proudfoot, M. Przybycien, A. Puri, P. Puzo, J. Qian, Y. Qin, A. Quadt, M. Queitsch-Maitland, A. Qureshi, P. Rados, F. Ragusa, G. Rahal, J. A. Raine, S. Rajagopalan, A. Ramirez Morales, T. Rashid, S. Raspopov, M. G. Ratti, D. M. Rauch, F. Rauscher, S. Rave, B. Ravina, I. Ravinovich, J. H. Rawling, M. Raymond, A. L. Read, N. P. Readioff, M. Reale, D. M. Rebuzzi, A. Redelbach, G. Redlinger, R. Reece, R. G. Reed, K. Reeves, L. Rehnisch, J. Reichert, A. Reiss, C. Rembser, H. Ren, M. Rescigno, S. Resconi, E. D. Resseguie, S. Rettie, E. Reynolds, O. L. Rezanova, P. Reznicek, E. Ricci, R. Richter, S. Richter, E. Richter-Was, O. Ricken, M. Ridel, P. Rieck, C. J. Riegel, O. Rifki, M. Rijssenbeek, A. Rimoldi, M. Rimoldi, L. Rinaldi, G. Ripellino, B. Ristić, E. Ritsch, I. Riu, J. C. Rivera Vergara, F. Rizatdinova, E. Rizvi, C. Rizzi, R. T. Roberts, S. H. Robertson, D. Robinson, J. E. M. Robinson, A. Robson, E. Rocco, C. Roda, Y. Rodina, S. Rodriguez Bosca, A. Rodriguez Perez, D. Rodriguez Rodriguez, A. M. Rodríguez Vera, S. Roe, C. S. Rogan, O. Røhne, R. Röhrig, C. P. A. Roland, J. Roloff, A. Romaniouk, M. Romano, N. Rompotis, M. Ronzani, L. Roos, S. Rosati, K. Rosbach, P. Rose, N.-A. Rosien, E. Rossi, E. Rossi, L. P. Rossi, L. Rossini, J. H. N. Rosten, R. Rosten, M. Rotaru, J. Rothberg, D. Rousseau, D. Roy, A. Rozanov, Y. Rozen, X. Ruan, F. Rubbo, F. Rühr, A. Ruiz-Martinez, Z. Rurikova, N. A. Rusakovich, H. L. Russell, J. P. Rutherfoord, E. M. Rüttinger, Y. F. Ryabov, M. Rybar, G. Rybkin, S. Ryu, A. Ryzhov, G. F. Rzehorz, P. Sabatini, G. Sabato, S. Sacerdoti, H. F.-W. Sadrozinski, R. Sadykov, F. Safai Tehrani, P. Saha, M. Sahinsoy, A. Sahu, M. Saimpert, M. Saito, T. Saito, H. Sakamoto, A. Sakharov, D. Salamani, G. Salamanna, J. E. Salazar Loyola, D. Salek, P. H. Sales De Bruin, D. Salihagic, A. Salnikov, J. Salt, D. Salvatore, F. Salvatore, A. Salvucci, A. Salzburger, J. Samarati, D. Sammel, D. Sampsonidis, D. Sampsonidou, J. Sánchez, A. Sanchez Pineda, H. Sandaker, C. O. Sander, M. Sandhoff, C. Sandoval, D. P. C. Sankey, M. Sannino, Y. Sano, A. Sansoni, C. Santoni, H. Santos, I. Santoyo Castillo, A. Santra, A. Sapronov, J. G. Saraiva, O. Sasaki, K. Sato, E. Sauvan, P. Savard, N. Savic, R. Sawada, C. Sawyer, L. Sawyer, C. Sbarra, A. Sbrizzi, T. Scanlon, J. Schaarschmidt, P. Schacht, B. M. Schachtner, D. Schaefer, L. Schaefer, J. Schaeffer, S. Schaepe, U. Schäfer, A. C. Schaffer, D. Schaile, R. D. Schamberger, N. Scharmberg, V. A. Schegelsky, D. Scheirich, F. Schenck, M. Schernau, C. Schiavi, S. Schier, L. K. Schildgen, Z. M. Schillaci, E. J. Schioppa, M. Schioppa, K. E. Schleicher, S. Schlenker, K. R. Schmidt-Sommerfeld, K. Schmieden, C. Schmitt, S. Schmitt, S. Schmitz, J. C. Schmoeckel, U. Schnoor, L. Schoeffel, A. Schoening, E. Schopf, M. Schott, J. F. P. Schouwenberg, J. Schovancova, S. Schramm, A. Schulte, H.-C. Schultz-Coulon, M. Schumacher, B. A. Schumm, Ph. Schune, A. Schwartzman, T. A. Schwarz, H. Schweiger, Ph. Schwemling, R. Schwienhorst, A. Sciandra, G. Sciolla, M. Scornajenghi, F. Scuri, F. Scutti, L. M. Scyboz, J. Searcy, C. D. Sebastiani, P. Seema, S. C. Seidel, A. Seiden, T. Seiss, J. M. Seixas, G. Sekhniaidze, K. Sekhon, S. J. Sekula, N. Semprini-Cesari, S. Sen, S. Senkin, C. Serfon, L. Serin, L. Serkin, M. Sessa, H. Severini, F. Sforza, A. Sfyrla, E. Shabalina, J. D. Shahinian, N. W. Shaikh, L. Y. Shan, R. Shang, J. T. Shank, M. Shapiro, A. S. Sharma, A. Sharma, P. B. Shatalov, K. Shaw, S. M. Shaw, A. Shcherbakova, Y. Shen, N. Sherafati, A. D. Sherman, P. Sherwood, L. Shi, S. Shimizu, C. O. Shimmin, M. Shimojima, I. P. J. Shipsey, S. Shirabe, M. Shiyakova, J. Shlomi, A. Shmeleva, D. Shoaleh Saadi, M. J. Shochet, S. Shojaii, D. R. Shope, S. Shrestha, E. Shulga, P. Sicho, A. M. Sickles, P. E. Sidebo, E. Sideras Haddad, O. Sidiropoulou, A. Sidoti, F. Siegert, Dj. Sijacki, J. Silva, M. Silva, M. V. Silva Oliveira, S. B. Silverstein, L. Simic, S. Simion, E. Simioni, M. Simon, R. Simoniello, P. Sinervo, N. B. Sinev, M. Sioli, G. Siragusa, I. Siral, S. Yu. Sivoklokov, J. Sjölin, P. Skubic, M. Slater, T. Slavicek, M. Slawinska, K. Sliwa, R. Slovak, V. Smakhtin, B. H. Smart, J. Smiesko, N. Smirnov, S. Yu. Smirnov, Y. Smirnov, L. N. Smirnova, O. Smirnova, J. W. Smith, M. N. K. Smith, M. Smizanska, K. Smolek, A. Smykiewicz, A. A. Snesarev, I. M. Snyder, S. Snyder, R. Sobie, A. M. Soffa, A. Soffer, A. Søgaard, D. A. Soh, G. Sokhrannyi, C. A. Solans Sanchez, M. Solar, E. Yu. Soldatov, U. Soldevila, A. A. Solodkov, A. Soloshenko, O. V. Solovyanov, V. Solovyev, P. Sommer, H. Son, W. Song, W. Y. Song, A. Sopczak, F. Sopkova, D. Sosa, C. L. Sotiropoulou, S. Sottocornola, R. Soualah, A. M. Soukharev, D. South, B. C. Sowden, S. Spagnolo, M. Spalla, M. Spangenberg, F. Spanò, D. Sperlich, F. Spettel, T. M. Spieker, R. Spighi, G. Spigo, L. A. Spiller, D. P. Spiteri, M. Spousta, A. Stabile, R. Stamen, S. Stamm, E. Stanecka, R. W. Stanek, C. Stanescu, B. Stanislaus, M. M. Stanitzki, B. Stapf, S. Stapnes, E. A. Starchenko, G. H. Stark, J. Stark, S. H Stark, P. Staroba, P. Starovoitov, S. Stärz, R. Staszewski, M. Stegler, P. Steinberg, B. Stelzer, H. J. Stelzer, O. Stelzer-Chilton, H. Stenzel, T. J. Stevenson, G. A. Stewart, M. C. Stockton, G. Stoicea, P. Stolte, S. Stonjek, A. Straessner, J. Strandberg, S. Strandberg, M. Strauss, P. Strizenec, R. Ströhmer, D. M. Strom, R. Stroynowski, A. Strubig, S. A. Stucci, B. Stugu, J. Stupak, N. A. Styles, D. Su, J. Su, S. Suchek, Y. Sugaya, M. Suk, V. V. Sulin, D. M. S. Sultan, S. Sultansoy, T. Sumida, S. Sun, X. Sun, K. Suruliz, C. J. E. Suster, M. R. Sutton, S. Suzuki, M. Svatos, M. Swiatlowski, S. P. Swift, A. Sydorenko, I. Sykora, T. Sykora, D. Ta, K. Tackmann, J. Taenzer, A. Taffard, R. Tafirout, E. Tahirovic, N. Taiblum, H. Takai, R. Takashima, E. H. Takasugi, K. Takeda, T. Takeshita, Y. Takubo, M. Talby, A. A. Talyshev, J. Tanaka, M. Tanaka, R. Tanaka, B. B. Tannenwald, S. Tapia Araya, S. Tapprogge, A. Tarek Abouelfadl Mohamed, S. Tarem, G. Tarna, G. F. Tartarelli, P. Tas, M. Tasevsky, T. Tashiro, E. Tassi, A. Tavares Delgado, Y. Tayalati, A. C. Taylor, A. J. Taylor, G. N. Taylor, P. T. E. Taylor, W. Taylor, A. S. Tee, P. Teixeira-Dias, H. Ten Kate, P. K. Teng, J. J. Teoh, F. Tepel, S. Terada, K. Terashi, J. Terron, S. Terzo, M. Testa, R. J. Teuscher, S. J. Thais, T. Theveneaux-Pelzer, F. Thiele, D. W. Thomas, J. P. Thomas, A. S. Thompson, P. D. Thompson, L. A. Thomsen, E. Thomson, Y. Tian, R. E. Ticse Torres, V. O. Tikhomirov, Yu. A. Tikhonov, S. Timoshenko, P. Tipton, S. Tisserant, K. Todome, S. Todorova-Nova, S. Todt, J. Tojo, S. Tokár, K. Tokushuku, E. Tolley, K. G. Tomiwa, M. Tomoto, L. Tompkins, K. Toms, B. Tong, P. Tornambe, E. Torrence, H. Torres, E. Torró Pastor, C. Tosciri, J. Toth, F. Touchard, D. R. Tovey, C. J. Treado, T. Trefzger, F. Tresoldi, A. Tricoli, I. M. Trigger, S. Trincaz-Duvoid, M. F. Tripiana, W. Trischuk, B. Trocmé, A. Trofymov, C. Troncon, M. Trovatelli, F. Trovato, L. Truong, M. Trzebinski, A. Trzupek, F. Tsai, J. C.-L. Tseng, P. V. Tsiareshka, A. Tsirigotis, N. Tsirintanis, V. Tsiskaridze, E. G. Tskhadadze, I. I. Tsukerman, V. Tsulaia, S. Tsuno, D. Tsybychev, Y. Tu, A. Tudorache, V. Tudorache, T. T. Tulbure, A. N. Tuna, S. Turchikhin, D. Turgeman, I. Turk Cakir, R. Turra, P. M. Tuts, E. Tzovara, G. Ucchielli, I. Ueda, M. Ughetto, F. Ukegawa, G. Unal, A. Undrus, G. Unel, F. C. Ungaro, Y. Unno, K. Uno, J. Urban, P. Urquijo, P. Urrejola, G. Usai, J. Usui, L. Vacavant, V. Vacek, B. Vachon, K. O. H. Vadla, A. Vaidya, C. Valderanis, E. Valdes Santurio, M. Valente, S. Valentinetti, A. Valero, L. Valéry, R. A. Vallance, A. Vallier, J. A. Valls Ferrer, T. R. Van Daalen, W. Van Den Wollenberg, H. Van der Graaf, P. Van Gemmeren, J. Van Nieuwkoop, I. Van Vulpen, M. Vanadia, W. Vandelli, A. Vaniachine, P. Vankov, R. Vari, E. W. Varnes, C. Varni, T. Varol, D. Varouchas, K. E. Varvell, G. A. Vasquez, J. G. Vasquez, F. Vazeille, D. Vazquez Furelos, T. Vazquez Schroeder, J. Veatch, V. Vecchio, L. M. Veloce, F. Veloso, S. Veneziano, A. Ventura, M. Venturi, N. Venturi, V. Vercesi, M. Verducci, C. M. Vergel Infante, W. Verkerke, A. T. Vermeulen, J. C. Vermeulen, M. C. Vetterli, N. Viaux Maira, M. Vicente Barreto Pinto, I. Vichou, T. Vickey, O. E. Vickey Boeriu, G. H. A. Viehhauser, S. Viel, L. Vigani, M. Villa, M. Villaplana Perez, E. Vilucchi, M. G. Vincter, V. B. Vinogradov, A. Vishwakarma, C. Vittori, I. Vivarelli, S. Vlachos, M. Vogel, P. Vokac, G. Volpi, S. E. von Buddenbrock, E. Von Toerne, V. Vorobel, K. Vorobev, M. Vos, J. H. Vossebeld, N. Vranjes, M. Vranjes Milosavljevic, V. Vrba, M. Vreeswijk, T. Šfiligoj, R. Vuillermet, I. Vukotic, T. Ženiš, L. Živković, P. Wagner, W. Wagner, J. Wagner-Kuhr, H. Wahlberg, S. Wahrmund, K. Wakamiya, V. M. Walbrecht, J. Walder, R. Walker, S. D. Walker, W. Walkowiak, V. Wallangen, A. M. Wang, C. Wang, F. Wang, H. Wang, H. Wang, J. Wang, J. Wang, P. Wang, Q. Wang, R.-J. Wang, R. Wang, R. Wang, S. M. Wang, W. T. Wang, W. Wang, W. X. Wang, Y. Wang, Z. Wang, C. Wanotayaroj, A. Warburton, C. P. Ward, D. R. Wardrope, A. Washbrook, P. M. Watkins, A. T. Watson, M. F. Watson, G. Watts, S. Watts, B. M. Waugh, A. F. Webb, S. Webb, C. Weber, M. S. Weber, S. A. Weber, S. M. Weber, A. R. Weidberg, B. Weinert, J. Weingarten, M. Weirich, C. Weiser, P. S. Wells, T. Wenaus, T. Wengler, S. Wenig, N. Wermes, M. D. Werner, P. Werner, M. Wessels, T. D. Weston, K. Whalen, N. L. Whallon, A. M. Wharton, A. S. White, A. White, M. J. White, R. White, D. Whiteson, B. W. Whitmore, F. J. Wickens, W. Wiedenmann, M. Wielers, C. Wiglesworth, L. A. M. Wiik-Fuchs, A. Wildauer, F. Wilk, H. G. Wilkens, L. J. Wilkins, H. H. Williams, S. Williams, C. Willis, S. Willocq, J. A. Wilson, I. Wingerter-Seez, E. Winkels, F. Winklmeier, O. J. Winston, B. T. Winter, M. Wittgen, M. Wobisch, A. Wolf, T. M. H. Wolf, R. Wolff, M. W. Wolter, H. Wolters, V. W. S. Wong, N. L. Woods, S. D. Worm, B. K. Wosiek, K. W. Woźniak, K. Wraight, M. Wu, S. L. Wu, X. Wu, Y. Wu, T. R. Wyatt, B. M. Wynne, S. Xella, Z. Xi, L. Xia, D. Xu, H. Xu, L. Xu, T. Xu, W. Xu, B. Yabsley, S. Yacoob, K. Yajima, D. P. Yallup, D. Yamaguchi, Y. Yamaguchi, A. Yamamoto, T. Yamanaka, F. Yamane, M. Yamatani, T. Yamazaki, Y. Yamazaki, Z. Yan, H. J. Yang, H. T. Yang, S. Yang, Y. Yang, Z. Yang, W.-M. Yao, Y. C. Yap, Y. Yasu, E. Yatsenko, J. Ye, S. Ye, I. Yeletskikh, E. Yigitbasi, E. Yildirim, K. Yorita, K. Yoshihara, C. J. S. Young, C. Young, J. Yu, J. Yu, X. Yue, S. P. Y. Yuen, B. Zabinski, G. Zacharis, E. Zaffaroni, R. Zaidan, A. M. Zaitsev, T. Zakareishvili, N. Zakharchuk, J. Zalieckas, S. Zambito, D. Zanzi, D. R. Zaripovas, S. V. Zeißner, C. Zeitnitz, G. Zemaityte, J. C. Zeng, Q. Zeng, O. Zenin, D. Zerwas, M. Zgubič, D. F. Zhang, D. Zhang, F. Zhang, G. Zhang, H. Zhang, J. Zhang, L. Zhang, L. Zhang, M. Zhang, P. Zhang, R. Zhang, R. Zhang, X. Zhang, Y. Zhang, Z. Zhang, P. Zhao, X. Zhao, Y. Zhao, Z. Zhao, A. Zhemchugov, B. Zhou, C. Zhou, L. Zhou, M. S. Zhou, M. Zhou, N. Zhou, Y. Zhou, C. G. Zhu, H. L. Zhu, H. Zhu, J. Zhu, Y. Zhu, X. Zhuang, K. Zhukov, V. Zhulanov, A. Zibell, D. Zieminska, N. I. Zimine, S. Zimmermann, Z. Zinonos, M. Zinser, M. Ziolkowski, G. Zobernig, A. Zoccoli, K. Zoch, T. G. Zorbas, R. Zou, M. Zur Nedden, L. Zwalinski, ATLAS Collaboration

**Affiliations:** 1 Department of Physics, University of Adelaide, Adelaide, Australia; 2 Physics Department, SUNY Albany, Albany, NY USA; 3 Department of Physics, University of Alberta, Edmonton, AB Canada; 4 Department of Physics, Ankara University, Ankara, Turkey; 5 Istanbul Aydin University, Istanbul, Turkey; 6 Division of Physics, TOBB University of Economics and Technology, Ankara, Turkey; 7 LAPP, Université Grenoble Alpes, Université Savoie Mont Blanc, CNRS/IN2P3, Annecy, France; 8 High Energy Physics Division, Argonne National Laboratory, Argonne, IL USA; 9 Department of Physics, University of Arizona, Tucson, AZ USA; 10 Department of Physics, University of Texas at Arlington, Arlington, TX USA; 11 Physics Department, National and Kapodistrian University of Athens, Athens, Greece; 12 Physics Department, National Technical University of Athens, Zografou, Greece; 13 Department of Physics, University of Texas at Austin, Austin, TX USA; 14 Faculty of Engineering and Natural Sciences, Bahcesehir University, Istanbul, Turkey; 15 Faculty of Engineering and Natural Sciences, Istanbul Bilgi University, Istanbul, Turkey; 16 Department of Physics, Bogazici University, Istanbul, Turkey; 17 Department of Physics Engineering, Gaziantep University, Gaziantep, Turkey; 18 Institute of Physics, Azerbaijan Academy of Sciences, Baku, Azerbaijan; 19 Institut de Física d’Altes Energies (IFAE), Barcelona Institute of Science and Technology, Barcelona, Spain; 20 Institute of High Energy Physics, Chinese Academy of Sciences, Beijing, China; 21 Physics Department, Tsinghua University, Beijing, China; 22 Department of Physics, Nanjing University, Nanjing, China; 23 University of Chinese Academy of Science (UCAS), Beijing, China; 24 Institute of Physics, University of Belgrade, Belgrade, Serbia; 25 Department for Physics and Technology, University of Bergen, Bergen, Norway; 26 Physics Division, Lawrence Berkeley National Laboratory and University of California, Berkeley, CA USA; 27 Institut für Physik, Humboldt Universität zu Berlin, Berlin, Germany; 28 Albert Einstein Center for Fundamental Physics and Laboratory for High Energy Physics, University of Bern, Bern, Switzerland; 29 School of Physics and Astronomy, University of Birmingham, Birmingham, UK; 30 Centro de Investigaciónes, Universidad Antonio Nariño, Bogotá, Colombia; 31 Dipartimento di Fisica e Astronomia, Università di Bologna, Bologna, Italy; 32 INFN Sezione di Bologna, Bologna, Italy; 33 Physikalisches Institut, Universität Bonn, Bonn, Germany; 34 Department of Physics, Boston University, Boston, MA USA; 35 Department of Physics, Brandeis University, Waltham, MA USA; 36 Transilvania University of Brasov, Brasov, Romania; 37 Horia Hulubei National Institute of Physics and Nuclear Engineering, Bucharest, Romania; 38 Department of Physics, Alexandru Ioan Cuza University of Iasi, Iasi, Romania; 39 Physics Department, National Institute for Research and Development of Isotopic and Molecular Technologies, Cluj-Napoca, Romania; 40 University Politehnica Bucharest, Bucharest, Romania; 41 West University in Timisoara, Timisoara, Romania; 42 Faculty of Mathematics, Physics and Informatics, Comenius University, Bratislava, Slovakia; 43 Department of Subnuclear Physics, Institute of Experimental Physics of the Slovak Academy of Sciences, Kosice, Slovak Republic; 44 Physics Department, Brookhaven National Laboratory, Upton, NY USA; 45 Departamento de Física, Universidad de Buenos Aires, Buenos Aires, Argentina; 46 Cavendish Laboratory, University of Cambridge, Cambridge, UK; 47 Department of Physics, University of Cape Town, Cape Town, South Africa; 48 Department of Mechanical Engineering Science, University of Johannesburg, Johannesburg, South Africa; 49 School of Physics, University of the Witwatersrand, Johannesburg, South Africa; 50 Department of Physics, Carleton University, Ottawa, ON Canada; 51 Faculté des Sciences Ain Chock, Réseau Universitaire de Physique des Hautes Energies-Université Hassan II, Casablanca, Morocco; 52 Centre National de l’Energie des Sciences Techniques Nucleaires (CNESTEN), Rabat, Morocco; 53 Faculté des Sciences Semlalia, Université Cadi Ayyad, LPHEA-Marrakech, Marrakech, Morocco; 54 Faculté des Sciences, Université Mohamed Premier and LPTPM, Oujda, Morocco; 55 Faculté des sciences, Université Mohammed V, Rabat, Morocco; 56 CERN, Geneva, Switzerland; 57 Enrico Fermi Institute, University of Chicago, Chicago, IL USA; 58 LPC, Université Clermont Auvergne, CNRS/IN2P3, Clermont-Ferrand, France; 59 Nevis Laboratory, Columbia University, Irvington, NY USA; 60 Niels Bohr Institute, University of Copenhagen, Copenhagen, Denmark; 61 Dipartimento di Fisica, Università della Calabria, Rende, Italy; 62 INFN Gruppo Collegato di Cosenza, Laboratori Nazionali di Frascati, Frascati, Italy; 63 Physics Department, Southern Methodist University, Dallas, TX USA; 64 Physics Department, University of Texas at Dallas, Richardson, TX USA; 65 Department of Physics, Stockholm University, Stockholm, Sweden; 66 Oskar Klein Centre, Stockholm, Sweden; 67 Deutsches Elektronen-Synchrotron DESY, Hamburg and Zeuthen, Germany; 68 Lehrstuhl für Experimentelle Physik IV, Technische Universität Dortmund, Dortmund, Germany; 69 Institut für Kern- und Teilchenphysik, Technische Universität Dresden, Dresden, Germany; 70 Department of Physics, Duke University, Durham, NC USA; 71 SUPA-School of Physics and Astronomy, University of Edinburgh, Edinburgh, UK; 72 INFN e Laboratori Nazionali di Frascati, Frascati, Italy; 73 Physikalisches Institut, Albert-Ludwigs-Universität Freiburg, Freiburg, Germany; 74 II. Physikalisches Institut, Georg-August-Universität Göttingen, Göttingen, Germany; 75 Département de Physique Nucléaire et Corpusculaire, Université de Genève, Geneva, Switzerland; 76 Dipartimento di Fisica, Università di Genova, Genoa, Italy; 77 INFN Sezione di Genova, Genoa, Italy; 78 II. Physikalisches Institut, Justus-Liebig-Universität Giessen, Giessen, Germany; 79 SUPA-School of Physics and Astronomy, University of Glasgow, Glasgow, UK; 80 LPSC, Université Grenoble Alpes, CNRS/IN2P3, Grenoble INP, Grenoble, France; 81 Laboratory for Particle Physics and Cosmology, Harvard University, Cambridge, MA USA; 82 Department of Modern Physics and State Key Laboratory of Particle Detection and Electronics, University of Science and Technology of China, Hefei, China; 83 Institute of Frontier and Interdisciplinary Science and Key Laboratory of Particle Physics and Particle Irradiation (MOE), Shandong University, Qingdao, China; 84 School of Physics and Astronomy, Shanghai Jiao Tong University, KLPPAC-MoE, SKLPPC, Shanghai, China; 85 Tsung-Dao Lee Institute, Shanghai, China; 86 Kirchhoff-Institut für Physik, Ruprecht-Karls-Universität Heidelberg, Heidelberg, Germany; 87 Physikalisches Institut, Ruprecht-Karls-Universität Heidelberg, Heidelberg, Germany; 88 Faculty of Applied Information Science, Hiroshima Institute of Technology, Hiroshima, Japan; 89 Department of Physics, Chinese University of Hong Kong, Shatin, NT Hong Kong; 90 Department of Physics, University of Hong Kong, Hong Kong, China; 91 Department of Physics and Institute for Advanced Study, Hong Kong University of Science and Technology, Clear Water Bay, Kowloon, Hong Kong, China; 92 Department of Physics, National Tsing Hua University, Hsinchu, Taiwan; 93 Department of Physics, Indiana University, Bloomington, IN USA; 94 INFN Gruppo Collegato di Udine, Sezione di Trieste, Udine, Italy; 95 ICTP, Trieste, Italy; 96 Dipartimento di Chimica, Fisica e Ambiente, Università di Udine, Udine, Italy; 97 INFN Sezione di Lecce, Lecce, Italy; 98 Dipartimento di Matematica e Fisica, Università del Salento, Lecce, Italy; 99 INFN Sezione di Milano, Milan, Italy; 100 Dipartimento di Fisica, Università di Milano, Milan, Italy; 101 INFN Sezione di Napoli, Naples, Italy; 102 Dipartimento di Fisica, Università di Napoli, Naples, Italy; 103 INFN Sezione di Pavia, Pavia, Italy; 104 Dipartimento di Fisica, Università di Pavia, Pavia, Italy; 105 INFN Sezione di Pisa, Pisa, Italy; 106 Dipartimento di Fisica E. Fermi, Università di Pisa, Pisa, Italy; 107 INFN Sezione di Roma, Rome, Italy; 108 Dipartimento di Fisica, Sapienza Università di Roma, Rome, Italy; 109 INFN Sezione di Roma Tor Vergata, Rome, Italy; 110 Dipartimento di Fisica, Università di Roma Tor Vergata, Rome, Italy; 111 INFN Sezione di Roma Tre, Rome, Italy; 112 Dipartimento di Matematica e Fisica, Università Roma Tre, Rome, Italy; 113 INFN-TIFPA, Povo, Italy; 114 Università degli Studi di Trento, Trento, Italy; 115 Institut für Astro- und Teilchenphysik, Leopold-Franzens-Universität, Innsbruck, Austria; 116 University of Iowa, Iowa City, IA USA; 117 Department of Physics and Astronomy, Iowa State University, Ames, IA USA; 118 Joint Institute for Nuclear Research, Dubna, Russia; 119 Departamento de Engenharia Elétrica, Universidade Federal de Juiz de Fora (UFJF), Juiz de Fora, Brazil; 120 Universidade Federal do Rio De Janeiro COPPE/EE/IF, Rio de Janeiro, Brazil; 121 Universidade Federal de São João del Rei (UFSJ), São João del Rei, Brazil; 122 Instituto de Física, Universidade de São Paulo, São Paulo, Brazil; 123 KEK, High Energy Accelerator Research Organization, Tsukuba, Japan; 124 Graduate School of Science, Kobe University, Kobe, Japan; 125 Faculty of Physics and Applied Computer Science, AGH University of Science and Technology, Kraków, Poland; 126 Marian Smoluchowski Institute of Physics, Jagiellonian University, Kraków, Poland; 127 Institute of Nuclear Physics Polish Academy of Sciences, Kraków, Poland; 128 Faculty of Science, Kyoto University, Kyoto, Japan; 129 Kyoto University of Education, Kyoto, Japan; 130 Research Center for Advanced Particle Physics and Department of Physics, Kyushu University, Fukuoka, Japan; 131 Instituto de Física La Plata, Universidad Nacional de La Plata and CONICET, La Plata, Argentina; 132 Physics Department, Lancaster University, Lancaster, UK; 133 Oliver Lodge Laboratory, University of Liverpool, Liverpool, UK; 134 Department of Experimental Particle Physics, Jožef Stefan Institute and Department of Physics, University of Ljubljana, Ljubljana, Slovenia; 135 School of Physics and Astronomy, Queen Mary University of London, London, UK; 136 Department of Physics, Royal Holloway University of London, Egham, UK; 137 Department of Physics and Astronomy, University College London, London, UK; 138 Louisiana Tech University, Ruston, LA USA; 139 Fysiska institutionen, Lunds universitet, Lund, Sweden; 140 Centre de Calcul de l’Institut National de Physique Nucléaire et de Physique des Particules (IN2P3), Villeurbanne, France; 141 Departamento de Física Teorica C-15 and CIAFF, Universidad Autónoma de Madrid, Madrid, Spain; 142 Institut für Physik, Universität Mainz, Mainz, Germany; 143 School of Physics and Astronomy, University of Manchester, Manchester, UK; 144 CPPM, Aix-Marseille Université, CNRS/IN2P3, Marseille, France; 145 Department of Physics, University of Massachusetts, Amherst, MA USA; 146 Department of Physics, McGill University, Montreal, QC Canada; 147 School of Physics, University of Melbourne, Parkville, VIC Australia; 148 Department of Physics, University of Michigan, Ann Arbor, MI USA; 149 Department of Physics and Astronomy, Michigan State University, East Lansing, MI USA; 150 B.I. Stepanov Institute of Physics, National Academy of Sciences of Belarus, Minsk, Belarus; 151 Research Institute for Nuclear Problems of Byelorussian State University, Minsk, Belarus; 152 Group of Particle Physics, University of Montreal, Montreal, QC Canada; 153 P.N. Lebedev Physical Institute of the Russian Academy of Sciences, Moscow, Russia; 154 Institute for Theoretical and Experimental Physics (ITEP), Moscow, Russia; 155 National Research Nuclear University MEPhI, Moscow, Russia; 156 D.V. Skobeltsyn Institute of Nuclear Physics, M.V. Lomonosov Moscow State University, Moscow, Russia; 157 Fakultät für Physik, Ludwig-Maximilians-Universität München, Munich, Germany; 158 Max-Planck-Institut für Physik (Werner-Heisenberg-Institut), Munich, Germany; 159 Nagasaki Institute of Applied Science, Nagasaki, Japan; 160 Graduate School of Science and Kobayashi-Maskawa Institute, Nagoya University, Nagoya, Japan; 161 Department of Physics and Astronomy, University of New Mexico, Albuquerque, NM USA; 162 Institute for Mathematics, Astrophysics and Particle Physics, Radboud University Nijmegen/Nikhef, Nijmegen, The Netherlands; 163 Nikhef National Institute for Subatomic Physics, University of Amsterdam, Amsterdam, The Netherlands; 164 Department of Physics, Northern Illinois University, De Kalb, IL USA; 165 Budker Institute of Nuclear Physics, SB RAS, Novosibirsk, Russia; 166 Novosibirsk State University, Novosibirsk, Russia; 167 Department of Physics, New York University, New York, NY USA; 168 Ohio State University, Columbus, OH USA; 169 Faculty of Science, Okayama University, Okayama, Japan; 170 Homer L. Dodge Department of Physics and Astronomy, University of Oklahoma, Norman, OK USA; 171 Department of Physics, Oklahoma State University, Stillwater, OK USA; 172 Palacký University, RCPTM, Joint Laboratory of Optics, Olomouc, Czech Republic; 173 Center for High Energy Physics, University of Oregon, Eugene, OR USA; 174 LAL, Université Paris-Sud, CNRS/IN2P3, Université Paris-Saclay, Orsay, France; 175 Graduate School of Science, Osaka University, Osaka, Japan; 176 Department of Physics, University of Oslo, Oslo, Norway; 177 Department of Physics, Oxford University, Oxford, UK; 178 LPNHE, Sorbonne Université, Paris Diderot Sorbonne Paris Cité, CNRS/IN2P3, Paris, France; 179 Department of Physics, University of Pennsylvania, Philadelphia, PA USA; 180 Konstantinov Nuclear Physics Institute of National Research Centre “Kurchatov Institute”, PNPI, St. Petersburg, Russia; 181 Department of Physics and Astronomy, University of Pittsburgh, Pittsburgh, PA USA; 182 Laboratório de Instrumentação e Física Experimental de Partículas-LIP, Lisbon, Portugal; 183 Departamento de Física, Faculdade de Ciências, Universidade de Lisboa, Lisbon, Portugal; 184 Departamento de Física, Universidade de Coimbra, Coimbra, Portugal; 185 Centro de Física Nuclear da Universidade de Lisboa, Lisbon, Portugal; 186 Departamento de Física, Universidade do Minho, Braga, Portugal; 187 Departamento de Física Teorica y del Cosmos, Universidad de Granada, Granada, Spain; 188 Dep Física and CEFITEC of Faculdade de Ciências e Tecnologia, Universidade Nova de Lisboa, Caparica, Portugal; 189 Institute of Physics, Academy of Sciences of the Czech Republic, Prague, Czech Republic; 190 Czech Technical University in Prague, Prague, Czech Republic; 191 Faculty of Mathematics and Physics, Charles University, Prague, Czech Republic; 192 State Research Center Institute for High Energy Physics, NRC KI, Protvino, Russia; 193 Particle Physics Department, Rutherford Appleton Laboratory, Didcot, UK; 194 IRFU, CEA, Université Paris-Saclay, Gif-sur-Yvette, France; 195 Santa Cruz Institute for Particle Physics, University of California Santa Cruz, Santa Cruz, CA USA; 196 Departamento de Física, Pontificia Universidad Católica de Chile, Santiago, Chile; 197 Departamento de Física, Universidad Técnica Federico Santa María, Valparaíso, Chile; 198 Department of Physics, University of Washington, Seattle, WA USA; 199 Department of Physics and Astronomy, University of Sheffield, Sheffield, UK; 200 Department of Physics, Shinshu University, Nagano, Japan; 201 Department Physik, Universität Siegen, Siegen, Germany; 202 Department of Physics, Simon Fraser University, Burnaby, BC Canada; 203 SLAC National Accelerator Laboratory, Stanford, CA USA; 204 Physics Department, Royal Institute of Technology, Stockholm, Sweden; 205 Departments of Physics and Astronomy, Stony Brook University, Stony Brook, NY USA; 206 Department of Physics and Astronomy, University of Sussex, Brighton, UK; 207 School of Physics, University of Sydney, Sydney, Australia; 208 Institute of Physics, Academia Sinica, Taipei, Taiwan; 209 E. Andronikashvili Institute of Physics, Iv. Javakhishvili Tbilisi State University, Tbilisi, Georgia; 210 High Energy Physics Institute, Tbilisi State University, Tbilisi, Georgia; 211 Department of Physics, Technion, Israel Institute of Technology, Haifa, Israel; 212 Raymond and Beverly Sackler School of Physics and Astronomy, Tel Aviv University, Tel Aviv, Israel; 213 Department of Physics, Aristotle University of Thessaloniki, Thessaloniki, Greece; 214 International Center for Elementary Particle Physics and Department of Physics, University of Tokyo, Tokyo, Japan; 215 Graduate School of Science and Technology, Tokyo Metropolitan University, Tokyo, Japan; 216 Department of Physics, Tokyo Institute of Technology, Tokyo, Japan; 217 Tomsk State University, Tomsk, Russia; 218 Department of Physics, University of Toronto, Toronto, ON Canada; 219 TRIUMF, Vancouver, BC Canada; 220 Department of Physics and Astronomy, York University, Toronto, ON Canada; 221 Division of Physics and Tomonaga Center for the History of the Universe, Faculty of Pure and Applied Sciences, University of Tsukuba, Tsukuba, Japan; 222 Department of Physics and Astronomy, Tufts University, Medford, MA USA; 223 Department of Physics and Astronomy, University of California Irvine, Irvine, CA USA; 224 Department of Physics and Astronomy, University of Uppsala, Uppsala, Sweden; 225 Department of Physics, University of Illinois, Urbana, IL USA; 226 Instituto de Física Corpuscular (IFIC), Centro Mixto Universidad de Valencia - CSIC, Valencia, Spain; 227 Department of Physics, University of British Columbia, Vancouver, BC Canada; 228 Department of Physics and Astronomy, University of Victoria, Victoria, BC Canada; 229 Fakultät für Physik und Astronomie, Julius-Maximilians-Universität Würzburg, Würzburg, Germany; 230 Department of Physics, University of Warwick, Coventry, UK; 231 Waseda University, Tokyo, Japan; 232 Department of Particle Physics, Weizmann Institute of Science, Rehovot, Israel; 233 Department of Physics, University of Wisconsin, Madison, WI USA; 234 Fakultät für Mathematik und Naturwissenschaften, Fachgruppe Physik, Bergische Universität Wuppertal, Wuppertal, Germany; 235 Department of Physics, Yale University, New Haven, CT USA; 236 Yerevan Physics Institute, Yerevan, Armenia; 237 CERN, 1211 Geneva 23, Switzerland

## Abstract

Measurements of the azimuthal anisotropy in lead–lead collisions at $\sqrt{s_{_\text {NN}}}$ = 5.02 TeV are presented using a data sample corresponding to 0.49 ${\mathrm {nb}}^{-1}$ integrated luminosity collected by the ATLAS experiment at the LHC in 2015. The recorded minimum-bias sample is enhanced by triggers for “ultra-central” collisions, providing an opportunity to perform detailed study of flow harmonics in the regime where the initial state is dominated by fluctuations. The anisotropy of the charged-particle azimuthal angle distributions is characterized by the Fourier coefficients, $v_{2}$–$v_{7}$, which are measured using the two-particle correlation, scalar-product and event-plane methods. The goal of the paper is to provide measurements of the differential as well as integrated flow harmonics $v_{n}$ over wide ranges of the transverse momentum, 0.5 $<p_{\mathrm{T}}<$ 60 GeV, the pseudorapidity, $|\eta |<$ 2.5, and the collision centrality 0–80%. Results from different methods are compared and discussed in the context of previous and recent measurements in Pb+Pb collisions at $\sqrt{s_{_\text {NN}}}$ = 2.76 $\mathrm{TeV}$ and 5.02 $\mathrm{TeV}$. In particular, the shape of the $p_{\mathrm{T}}$dependence of elliptic or triangular flow harmonics is observed to be very similar at different centralities after scaling the $v_{n}$ and $p_{\mathrm{T}}$values by constant factors over the centrality interval 0–60% and the $p_{\mathrm{T}}$range 0.5 $< p_{\mathrm{T}}<$ 5 GeV.

## Introduction

One of the primary goals of ultra-relativistic heavy-ion collisions is the study of the hot and dense medium formed there, usually referred to as the quark-gluon plasma (QGP) [1–5]. The existence of the QGP phase of nuclear matter has been confirmed by a wealth of experimental data [5, 6]. In particular, properties related to the collective expansion of the QGP (e.g. the equation of state [7] and shear viscosity [8]) are inferred from measurements of azimuthal anisotropies of produced particles. It is now understood that the azimuthal anisotropy results from large initial pressure gradients in the hot, dense matter created in the collisions [9, 10]. These pressure gradients transform the initial spatial anisotropies of nuclear collisions into momentum anisotropies of the final-state particle production, which are experimentally characterized by Fourier (flow) harmonics of the azimuthal angle distributions of produced particles. The discovery of large flow harmonics at RHIC, and more recently at much higher collision energy at the LHC [11–14], has significantly deepened the understanding of the QGP, as explored theoretically by the QCD lattice [15]. In particular, the recent measurements of azimuthal anisotropy help to constrain the commonly used modelling of the dynamics of heavy-ion collisions based on relativistic viscous hydrodynamics. Typically, in the hydrodynamic models, a strongly interacting quark–gluon medium is formed shortly after the collision and its evolution is well described by relativistic fluid dynamics [8]. Detailed investigations, based on hydrodynamics, have shown that the produced medium has properties similar to those of an almost ideal fluid characterized by a very low ratio of viscosity to entropy density, $\eta /s$. Precise azimuthal anisotropy measurements over a wide range in kinematic variables and centrality are key elements to improving our understanding of the strongly coupled QGP because of their unique sensitivity to $\eta /s$.

The azimuthal angular distribution of single produced particles can be expanded in a Fourier series [16, 17]:

1 $$\begin{aligned} \frac{\text {d}N}{\text {d}\phi } = \frac{N_{0}}{2\pi }\left( 1+ \sum _{n=1} 2v_{n} \cos \left[ n\left( \phi - \Phi _n\right) \right] \right) , \end{aligned}$$

where $$N_0$$ is the total particle yield, $$\phi $$ is the azimuthal angle of the produced particles and the $$v_{n} $$ and $$\Phi _n$$ are, respectively, the magnitude of the *n*th-order azimuthal anisotropy and the orientation of the *n*th-order symmetry plane. The $$v_{n}$$ coefficients – also called *flow harmonics* – are typically measured as a function of particle pseudorapidity^1^ ($$\eta $$), transverse momentum ($$p_{\mathrm{T}}$$), and the degree of overlap between the colliding nuclei (centrality). Event-by-event fluctuations in the number and position of the interacting nucleons give rise to anisotropic flow fluctuations [18].

The first harmonic, $v_{1}$, is known as *directed flow* and refers to the sideward motion of participants in ultra-relativistic nuclear collisions, and it carries information from the early stage of the collision. The most extensive studies are related to the second flow harmonic, $v_{2}$, also known as *elliptic flow*. Elliptic flow is sensitive to the initial spatial asymmetry of the almond-shaped overlapping zone of the colliding nuclei. The higher-order coefficients, $n>2$, are also important due to their sensitivity to initial-state geometric fluctuations and viscous effects [16–18].

During the first operational period at the LHC (Run 1) lead ions were collided at energy per colliding nucleon–nucleon pair $\sqrt{s_{_\text {NN}}}$ = 2.76 $\mathrm{TeV}$, which is about 13 times larger than the highest collision energy attained at RHIC in Au+Au collisions. ATLAS and other LHC experiments collected large samples of heavy-ion data enabling extensive studies of the elliptic flow and higher-order Fourier coefficients. ATLAS measurements of flow harmonics were performed in broad regions of transverse momentum, pseudorapidity and event centrality, using the standard event-plane (EP) method [12], two-particle correlations (2PC) [13] and multi-particle cumulants [19]. Significant (non-zero) flow harmonics up to $v_{6}$ were measured in Pb+Pb collisions at $\sqrt{s_{_\text {NN}}}$ = 2.76 $\mathrm{TeV}$, which provide important constraints on the bulk and shear viscosity of the QGP medium [20]. Additionally, by comparing RHIC (STAR [21] and PHENIX [22]) and LHC (ATLAS [12], ALICE [23] and CMS [24]) results, it was found that for similar centrality of Au+Au and Pb+Pb interactions, $v_{n}$ as a function of $p_{\mathrm{T}}$is approximately independent of collision energy. There is an initial rise of $v_{n}$ with $p_{\mathrm{T}}$up to about 3 $\mathrm{GeV}$ and then a drop-off at higher values of $p_{\mathrm{T}}$, and only weak dependence for $p_{\mathrm{T}}~> ~8{-}9~\mathrm{GeV}$. As a function of centrality, there is similarly little variation with collision energy. The second harmonic, $v_{2}$, exhibits the most pronounced centrality variation, rising to a maximum for mid-central collisions, and then falling off for the most central collisions, reflecting variations in the shape of the initial collision geometry. The harmonic, $v_{3}$, referred as triangular flow, which has a value similar to $v_{2}$ in central collisions, shows a weaker dependence on centrality, as do the higher-order harmonics.

At the start of the second operational period of the LHC (Run 2), in November and December of 2015, lead–lead collisions with higher collision energy per nucleon pair of $\sqrt{s_{_\text {NN}}}= 5.02$ $\mathrm{TeV}$ were collected by the LHC experiments. The goal of this paper is to present and discuss the first ATLAS measurements of $v_{n}$ harmonics at this energy, using the two-particle correlation [17], scalar-product (SP) [25] and event-plane [16, 17] methods. Comparing the 2PC and SP results can quantify the extent to which the two-particle correlations factorize into the product of the flow harmonics corresponding to single-particle angular distributions [26, 27]. While the SP and EP methods are expected to yield similar values of the $v_{n}$, small variations due to their different sensitivity to initial-state geometric fluctuations can nevertheless occur [28]. To study the energy dependence, the 2PC and EP flow harmonics are compared with previous ATLAS measurements in 2.76 $\mathrm{TeV}$ Pb+Pb collisions [12, 13]. The results presented in this paper, together with the results on azimuthal anisotropy from other LHC experiments [29, 30], provide further opportunity to study the properties of the QGP, constrain hydrodynamic models, study transport coefficients and extract the temperature dependence of transport coefficients, including $\eta /s$.

The organization of this paper is as follows. Section 2 gives a brief overview of the ATLAS detector and the subsystems that are used in this analysis. Section 3 describes the datasets, triggers and the offline selection criteria used to select events and charged-particle tracks. Section 4 gives details of the scalar-product, event-plane and two-particle correlation methods, which are used to measure the $v_{n}$. Section 5 describes the systematic uncertainties associated with the measured $v_{n}$. Section 6 presents the main results of the analysis, which are the $p_{\mathrm{T}}$, $\eta$ and centrality dependence of the $v_{n}$ and comparisons of results from the different methods. Section 7 gives a summary of the main results and observations.

## Experimental set-up

The measurements were performed using the ATLAS detector [31] at the LHC. The principal components used in this analysis are the inner detector (ID), minimum-bias trigger scintillators (MBTS), calorimeter, zero-degree calorimeters (ZDC), and the trigger and data acquisition systems. The ID detects charged particles within the pseudorapidity range $|\eta |~<~2.5$ using a combination of silicon pixel detectors, including the “insertable B-layer” [32, 33] that was installed between Run 1 and Run 2, silicon microstrip detectors (SCT), and a straw-tube transition radiation tracker (TRT), all immersed in a 2 T axial magnetic field [34]. The MBTS system detects charged particles over $2.07< |\eta | < 3.86$ using two scintillator-based hodoscopes on each side of the detector, positioned at $z=\pm 3.6~{\mathrm m}$. These hodoscopes were rebuilt between Run 1 and Run 2. The ATLAS calorimeter system consists of a liquid argon (LAr) electromagnetic (EM) calorimeter covering $|\eta |<3.2$, a steel–scintillator sampling hadronic calorimeter covering $|\eta |<1.7$, a LAr hadronic calorimeter covering $1.5<|\eta |<3.2$, and two LAr electromagnetic and hadronic forward calorimeters (FCal) covering $3.2<|\eta |<4.9$. The ZDC, situated at approximately $\pm 140$ m from the nominal IP, detect neutral particles, mostly neutrons and photons, with $|\eta |~>~8.3$. The ZDC use tungsten plates as absorbers, and quartz rods sandwiched between the tungsten plates as the active medium. The ATLAS trigger system [35] consists of a first-level (L1) trigger implemented using a combination of dedicated electronics and programmable logic, and a software-based high-level trigger.

## Event and track selection

The Pb+Pb dataset used in this paper corresponds to an integrated luminosity of 0.49 ${\mathrm {nb}}^{-1}$. Minimum-bias events were selected by two mutually exclusive triggers :

- Events with smaller impact parameter (semi-central and central collisions) were selected by a trigger that required the total transverse energy ($E_{\mathrm{T}}$) deposited in the calorimeters at L1 to be above 50 $\mathrm{GeV}$.
- Collisions with large impact parameter (peripheral events) were selected by a trigger that required the total transverse energy at L1 to be less than 50 $\mathrm{GeV}$, one neutron on either side in the ZDC ($|\eta | > 8.3$), and at least one reconstructed track in the ID.

The minimum-bias triggers sampled a total luminosity of 22 $\upmu {{\mathrm {b}}}^{-1}$. To enhance the statistics of ultra-central collisions, additional data samples were recorded by two dedicated triggers – UCC-1 and UCC-2 – that selected events in which the total $E_{\mathrm{T}}$ in the FCal at L1 was more than 4.21 $\mathrm{TeV}$ and 4.54 $\mathrm{TeV}$, respectively. The UCC-1 trigger sampled a luminosity of 45 $\upmu {\mathrm {b}}^{-1}$ while the UCC-2 trigger sampled the entire luminosity of 0.49 ${\mathrm {nb}}^{-1}$. The luminosities sampled by the different triggers are listed in Table 1.

**Table 1:** The luminosities sampled by the triggers used in the analysis

Trigger	Sampled luminosity
Minimum-bias	22 $\upmu {\mathrm {b}}^{-1}$
UCC-1	45 $\upmu {\mathrm {b}}^{-1}$
UCC-2	0.49 ${\mathrm {nb}}^{-1}$

In the offline analysis the *z* coordinate of the primary vertex [36] is required to be within 10 cm of the nominal interaction point. The fraction of events containing more than one inelastic interaction (pile-up) is estimated to be at the level of 0.2%. The fraction varies with $\Sigma E_\mathrm {T}^\mathrm {FCal}$, and for ultra-central collisions it amounts to a few percent. Pile-up events were removed by exploiting the correlations between the transverse energy measured in the FCal and in the ZDC as well as the number of tracks associated with the primary vertex, $N^{\mathrm {rec}}_{\mathrm {ch}}$. As the pile-up is very small, in a typical pile-up event the track multiplicity associated with the primary vertex belongs to a single Pb+Pb collision, while the energy deposited in calorimeters contains contributions from multiple, mostly two, collisions. Therefore, events with small values of $N^{\mathrm {rec}}_{\mathrm {ch}}$ and large $\Sigma E_\mathrm {T}^\mathrm {FCal}$ that differ markedly from those of the majority of Pb+Pb collisions are removed from the analysis [19]. In addition, the anti-correlation between the $\Sigma E_\mathrm {T}^\mathrm {FCal}$ and the number of neutrons detected in ZDC is also used to suppress pile-up events. Events with the number of neutrons (as recorded in the ZDC) much higher than the number expected from the bulk of events for a given value $\Sigma E_\mathrm {T}^\mathrm {FCal}$ are rejected.

**Fig. 1:**
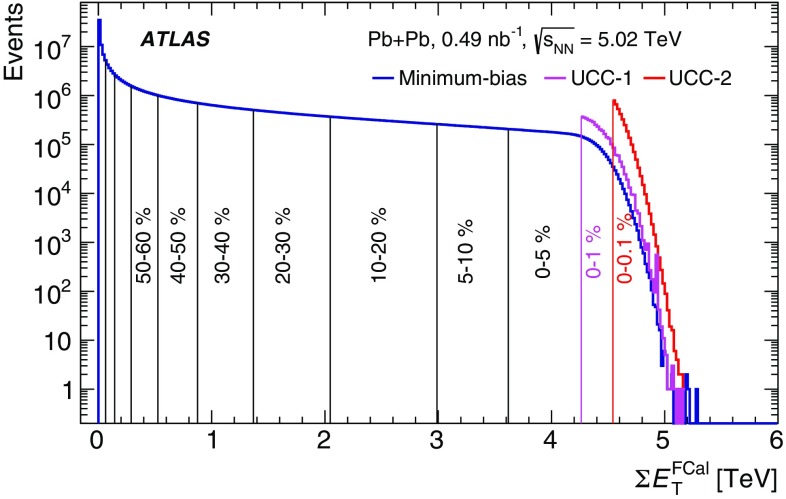
The $\Sigma E_{\mathrm{T}}^{\mathrm {FCal}}$ distribution in $\sqrt{s_{_\text {NN}}}$ = 5.02 $\mathrm{TeV}$ Pb+Pb data for events selected by the minimum-bias trigger. The $\Sigma E_{\mathrm{T}}^\mathrm {FCal}$ thresholds for several centrality intervals are marked with vertical lines and labelled on the plot. Also shown are the number of events over the 0–1% and 0–0.1% centrality intervals selected by the ultra-central triggers

The heavy-ion collision geometry is defined by its impact parameter, *b*. As the actual event-by-event impact parameter is not accessible experimentally, the centrality classification is based on the transverse energy measured in the forward calorimeter, $\Sigma E_\mathrm {T}^\mathrm {FCal}$, which exhibits a strong monotonic correlation with *b*. A model based on the Monte Carlo (MC) Glauber approach [37, 38] is used to obtain the mapping from the observed $\Sigma E_\mathrm {T}^\mathrm {FCal}$ to the primary properties, such as the number of binary nucleon–nucleon interactions, $N_{\mathrm {coll}}$, or the number of nucleons participating in the nuclear collision, $N_{\mathrm {part}}$, for each centrality interval. The Glauber model also provides a correspondence between the $\Sigma E_\mathrm {T}^\mathrm {FCal}$ distribution and the sampling fraction of the total inelastic Pb+Pb cross-section, allowing the setting of the centrality percentiles [12]. For this analysis a selection of the 80% most central collisions (i.e. centrality 0–80%) is used to avoid any diffractive, photonuclear, and other inelastic processes that contribute significantly to very peripheral collisions (centrality 80–100%). Additionally, the events selected by UCC-1 and UCC-2 are used only over the 0–1% and 0–0.1% centrality intervals, respectively. Figure 1 shows the distribution of $\Sigma E_\mathrm {T}^\mathrm {FCal}$ in the data, and thresholds for the selection of several centrality intervals. The correspondence of centrality intervals to $\langle N_{\mathrm {part}}\rangle$ values is provided in Table 2.

**Table 2:** The correspondence between centrality intervals used in the analysis and $\langle N_{\mathrm {part}} \rangle$ values

Centrality (%)	$\langle N_{\mathrm {part}} \rangle$	Centrality (%)	$\langle N_{\mathrm {part}} \rangle$	Centrality (%)	$\langle N_{\mathrm {part}} \rangle$
0–0.1	406.6 ± 1.3	10–20	264.1 ± 2.9	50–60	53.9 ± 2.0
0–1	402.9 ± 1.5	20–30	189.2 ± 2.8	60–70	30.6 ± 1.5
0–5	384.5 ± 1.9	30–40	131.4 ± 2.6	70–80	15.4 ± 1.0
5–10	333.1 ± 2.7	40–50	87.0 ± 2.4		

In order to study the performance of the ATLAS detector, a minimum-bias sample of 4M Pb+Pb MC events was generated using version 1.38b of HIJING [39]. The effect of flow was added after the generation using an “afterburner” [40] procedure in which the $p_{\mathrm{T}}$, $\eta$ and centrality dependence of the $v_{n}$, as measured in the $\sqrt{s_{_\text {NN}}}=2.76~\mathrm{TeV}$ Pb+Pb data [13], is implemented by artificially rearranging the $\phi$ positions of the generated particles. The generated sample was passed through a full simulation of the ATLAS detector using ${\textsc {geant}}$4 [41], and the simulated events are reconstructed using the same algorithms as used for real data. Charged-particle tracks are reconstructed from the signals in the ID. A reconstruction procedure developed for tracking in dense environments in $pp$collisions, and optimized for heavy-ion collisions, was used for this purpose [42]. In the analysis the set of reconstructed tracks is filtered using several selection criteria. The tracks are required to have $p_{\mathrm{T}}>0.5~\mathrm{GeV}$, $|\eta |<2.5$, at least two pixel hits, with the additional requirement of a hit in the first pixel layer when one is expected, at least eight SCT hits, and at most one missing hit in the SCT. A hit is expected if the extrapolated track crosses an active region of a pixel module that has not been disabled, and a hit is said to be missing when it is expected but not found. In addition, the transverse ($d_0$) and longitudinal ($z_0\sin \theta$) impact parameters of the track relative to the vertex are required to be less than 1 mm. The track-fit quality parameter $\chi ^2/$ndof is required to be less than 6.

The MC sample is used to determine the track-reconstruction efficiency as a function of $p_{\mathrm{T}}$, $\eta$ and centrality, $\epsilon (p_{\mathrm{T}},\eta , {\mathrm {centrality}})$. The efficiency is defined as the fraction of primary [36] charged particles matched to reconstructed tracks. The matching criterion is that the weighted fraction of hits in a reconstructed track originating from a given generated particle is above 30%. Different weights are assigned to pixel, SCT and TRT signals to be more robust against fake tracks, which are defined below. At mid-rapidity ($|\eta |<1$) and for events with centrality $<5\%$, the reconstruction efficiency is $\sim$ 60% at low $p_{\mathrm{T}}$ and increases to $\sim$ 75% at higher $p_{\mathrm{T}}$. For $|\eta |>1$ the efficiency decreases to about 40–60% depending on the $p_{\mathrm{T}}$and centrality. The reconstruction efficiency depends weakly on the centrality for low-$p_{\mathrm{T}}$ tracks, for which it is smaller in the most central events by about 5% as compared to mid-central and peripheral collisions. For tracks with $p_{\mathrm{T}}> 1~\mathrm{GeV}$ the dependence on centrality is less than 1%.

The fraction of tracks that are not matched to primary, generated MC particles or are produced from random combinations of hits in the ID, both referred to as “fake tracks”, is found to depend significantly on $\eta$. For $|\eta |<1$, it is $\sim$10% for low-$p_{\mathrm{T}}$ tracks in the most central 5% Pb+Pb events, and about 5% for more peripheral collisions. In the forward part of the detector, especially for $1<|\eta |<2$ where detector services reside, the fake rate can reach 18% for low $p_{\mathrm{T}}$tracks in the most central collisions. The fake rate drops rapidly for higher $p_{\mathrm{T}}$ and also decreases gradually towards more peripheral collisions. For $p_{\mathrm{T}}\ > 10~\mathrm{GeV}$ and 0–5% centrality it rises to about 5%.

## Analysis procedure

Three analysis techniques are used to determine the flow harmonics: the two-particle correlation method, which uses only the information from the tracking detectors, and the scalar-product and event-plane methods, which also use information from the FCal.

In all approaches the differential flow harmonics are first obtained in narrow intervals of $$p_{\mathrm{T}}$$, $$\eta $$ and centrality. Integrated quantities are obtained by taking into account the track reconstruction efficiency, $$\epsilon $$, and fake rate, *f*. A $$p_{\mathrm{T}}$$-, $$\eta $$- and centrality-dependent weight factor $$w = (1-f)/\epsilon $$ is applied to each track in the 2PC measurement and to scale each bin of the differential $$v_{n} $$ distributions in the SP and EP methods.

All analysis methods utilize the minimum-bias sample of 22 $\upmu {\mathrm {b}} ^{-1}$. In addition, the SP and EP analyses use the ultra-central samples of 45 $\upmu {\mathrm {b}} ^{-1}$ and 0.49 ${\mathrm {nb}}^{-1}$.

### Two-particle correlation analysis

The 2PC method has been used extensively by ATLAS for correlation measurements [13, 43–48]. In the 2PC method, the distribution of particle pairs in relative azimuthal angle $\Delta \phi =\phi ^a-\phi ^b$ and pseudorapidity separation $\Delta \eta =\eta ^a-\eta ^b$ is measured. Here the labels *a* and *b* denote the two particles used to make the pair. They are conventionally called the “trigger” and “associated” particles, respectively. In this analysis, the two particles are charged particles reconstructed by the ATLAS tracking system, over the full azimuth and $|\eta |<2.5$, resulting in a pair-acceptance coverage of $\pm 5.0$ units in $\Delta \eta$.

In order to account for the detector acceptance effects, the correlation is constructed from the ratio of the distribution in which the trigger and associated particles are taken from the same event to the distribution in which the trigger and associated particles are taken from two different events. These two distributions are referred to as the “same-event” (*S*) or “foreground” distribution and the “mixed-event” or “background” (*B*) distribution, respectively, and the ratio is written as:

$$\begin{aligned} C(\Delta \eta ,\Delta \phi ) =\frac{S(\Delta \phi ,\Delta \eta )}{B(\Delta \phi ,\Delta \eta )}. \end{aligned}$$

The same-event distribution includes both the physical correlations and correlations arising from detector acceptance effects. On the other hand, the mixed-event distribution reflects only the effects of detector inefficiencies and non-uniformity, but contains no physical correlations. To ensure that the acceptance effects in the *B* distribution match closely those in the *S* distribution, the *B* distribution is constructed from particles from two different events that have similar multiplicity and *z*-vertex. Furthermore, in order to account for the effects of tracking efficiency $\epsilon (p_{\mathrm{T}},\eta )$, and fakes $f(p_{\mathrm{T}},\eta )$, each pair is weighted by

$$\begin{aligned} w_{a,b} = \frac{ (1-f(p_{\mathrm {T}}^{a},\eta ^a))(1-f(p_{\mathrm {T}}^{b},\eta ^b))}{\epsilon (p_{\mathrm {T}}^{a},\eta ^a)\epsilon (p_{\mathrm {T}}^{b},\eta ^b)} \end{aligned}$$

for *S* and *B*. In the ratio *C*, the acceptance effects largely cancel out and only the physical correlations remain [49]. Typically, the two-particle correlations are used only to study the shape of the correlations in $\Delta \phi$, and are conveniently normalized. In this paper, the normalization of $C(\Delta \eta ,\Delta \phi )$ is chosen such that the $\Delta \phi$-averaged value of $C(\Delta \eta ,\Delta \phi )$ is unity for $|\Delta \eta |>2$.

**Fig. 2:**
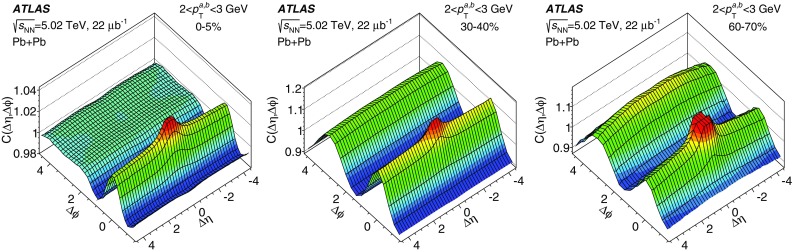
Two-particle correlation functions $C(\Delta \eta ,\Delta \phi )$ in 5.02 $\mathrm{TeV}$ Pb+Pb collisions for $2~<~p_{\mathrm {T}}^{a,b} <~3~\mathrm{GeV}$. The left, middle and right panels correspond to the 0–5%, 30–40% and 60–70% centrality classes, respectively. The distributions are truncated to suppress the peak at $\Delta \eta = \Delta \phi = 0$ to show the long-range correlations in greater detail

Figure 2 shows $C(\Delta \eta ,\Delta \phi )$ for several centrality intervals for $2~<~p_{\mathrm {T}}^{a,b} <~3~\mathrm{GeV}$. In all cases a peak is seen in the correlation at $(\Delta \eta ,\Delta \phi )\sim (0,0)$. This “near-side” peak arises from a range of sources including resonance decays, Hanbury Brown and Twiss (HBT) correlations [50] and jet fragmentation [51]. The long-range (large $|\Delta \eta |$) correlations are the result of the global anisotropy of the event and are the focus of the study in this paper.

To investigate the $\Delta \phi$ dependence of the long-range ($|\Delta \eta |~>~2$) correlation in more detail, the projection on to the $\Delta \phi$ axis is constructed as follows:

$$\begin{aligned} C(\Delta \phi ) = \frac{\int _{2}^{5} \text {d}|\Delta \eta | \; S(\Delta \phi ,|\Delta \eta |)}{\int _{2}^{5} \text {d}|\Delta \eta | \; B(\Delta \phi ,|\Delta \eta |)}\equiv \frac{S(\Delta \phi )}{B(\Delta \phi )}. \end{aligned}$$

The $|\Delta \eta |~>~2$ requirement is imposed to reject the near-side jet peak and focus on the long-range features of the correlation functions.

In a similar fashion to the single-particle distribution (Eq. (1)), the 2PC can be expanded as a Fourier series:

2 $$\begin{aligned} C(\Delta \phi ) =C_{0}\left( 1+\Sigma _{n=1}^{\infty }v_{n,n} (p_{\mathrm {T}}^{a},p_{\mathrm {T}}^{b}) \cos (n\Delta \phi )\right) , \end{aligned}$$

where the $v_{n,n}$ are the Fourier coefficients of the 2PC, and $C_0$ is its average value. If the two-particle distribution is simply the product of two single-particle distributions, then it can be shown that the Fourier coefficients of the 2PC factorize as [49]:

3 $$\begin{aligned} v_{n,n} (p_{\mathrm {T}}^{a},p_{\mathrm {T}}^{b})=v_{n} (p_{\mathrm {T}}^{a})v_{n} (p_{\mathrm {T}}^{b}).\ \end{aligned}$$

In Ref. [13] it was demonstrated that the factorization of $v_{n,n}$, given by Eq. (3), is valid in central and mid-central Pb+Pb collisions at $\sqrt{s_{NN}}$ = 2.76 GeV as long as one of the correlated particles is from a low $p_{\mathrm{T}}$range. A breakdown of the factorization is expected when the anisotropy does not arise from flow, e.g. in peripheral collisions at high $p_{\mathrm{T}}$. The factorization is also expected to break when the $\eta$ separation between the particles is small, and short-range correlations dominate [13]. However, the $|\Delta \eta |~>~2$ requirement removes most such short-range correlations. In the phase-space region where Eq. (3) holds, the $v_{n}$ ($p_{\mathrm {T}}^{b}$) can be evaluated from the measured $v_{n,n}$ as:

4 $$\begin{aligned} v_{n} (p_{\mathrm {T}}^{b})=\frac{v_{n,n} (p_{\mathrm {T}}^{a},p_{\mathrm {T}}^{b})}{v_{n} (p_{\mathrm {T}}^{a})}= \frac{v_{n,n} (p_{\mathrm {T}}^{a},p_{\mathrm {T}}^{b})}{\sqrt{v_{n,n} (p_{\mathrm {T}}^{a},p_{\mathrm {T}}^{a})}}, \end{aligned}$$

where $v_{n,n} (p_{\mathrm {T}}^{a},p_{\mathrm {T}}^{a})=v_{n} ^2(p_{\mathrm {T}}^{a})$ is used in the denominator. In this analysis, for most of the 2PC results the $v_{n}$ ($p_{\mathrm {T}}^{b}$) will be evaluated using Eq. (4) with $0.5~<~p_{\mathrm {T}}^{a} <~5.0~\mathrm{GeV}$. The lower cut-off of 0.5 $\mathrm{GeV}$ on $p_{\mathrm {T}}^{a}$ is the lower limit of $p_{\mathrm{T}}$measurements in this paper. The upper cut-off on $p_{\mathrm {T}}^{a}$ is chosen to exclude high-$p_{\mathrm{T}}$ particles, which predominantly come from jets and are not expected to obey Eq. (4).

**Fig. 3:**
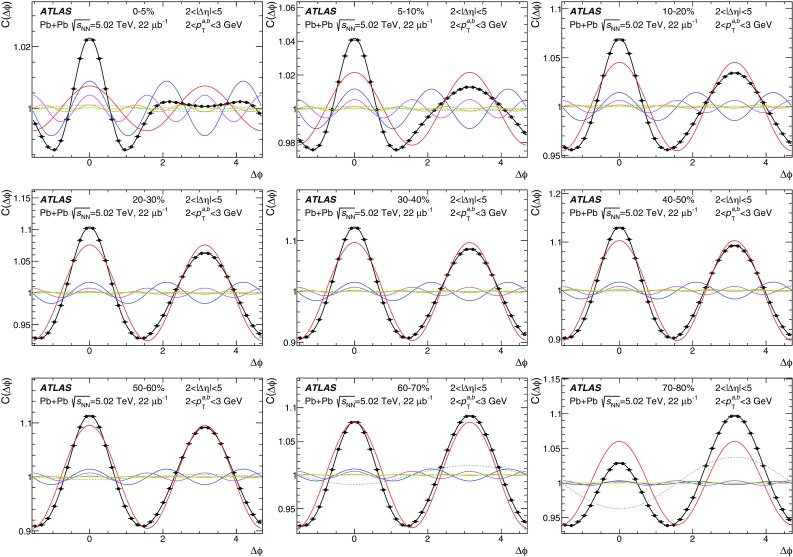
One-dimensional two-particle correlation functions $C(\Delta \phi )$ in 5.02 $\mathrm{TeV}$ Pb+Pb collisions for $2~<~p_{\mathrm {T}}^{a,b} ~<~3~\mathrm{GeV}$ (points). The solid-black line indicates a fit to Eq. (2) containing harmonics $v_{n,n}$ up to $n=6$. The dashed grey line shows the contribution of the $v_{1,1}$. The contributions of the $v_{2,2}$–$v_{6,6}$ are indicated by the coloured lines ($v_{2,2}$-red, $v_{3,3}$-blue, $v_{4,4}$-magenta, $v_{5,5}$-orange, $v_{6,6}$-green), and can be identified by the number of peaks that they have. Each panel corresponds to a different centrality class. The *y*-axis range for the different panels is different

Figure 3 shows one-dimensional 2PCs as a function of $\Delta \phi$ for $2<p_{\mathrm {T}}^{a,b} <3~\mathrm{GeV}$ and for several different centrality intervals. The correlations are normalized to have a mean value ($C_{0}$ in Eq. (2)) of 1.0. The continuous line in Fig. 3 is a Fourier fit to the correlation (Eq. (2)) that includes harmonics up to $n=6$. The contribution of the individual $v_{n,n}$ are also shown. The modulation in the correlation about its mean value is smallest in the most central events (top left panel) and increases towards mid-central events, reaching a maximum in the 40–50% centrality interval and then decreases. In central collisions, the $v_{2,2}$–$v_{4,4}$ are of comparable magnitude. But for other centralities, where the average collision geometry is elongated, the $v_{2,2}$ is significantly larger than the other $v_{n,n}$ for $n\ge 3$. In the central events the “away-side” peak at $\Delta \phi \sim \pi$ is also much broader because all the significant harmonics are of similar magnitude, while in mid-central events the near-side and away-side peaks are quite symmetric as the $v_{2,2}$ dominates. In central and mid-central events, the near-side peak is larger than the away-side peak. However, for the 60–70% and more peripheral centralities, the away-side peak becomes larger due to the presence of a large negative $v_{1,1}$ component. This negative $v_{1,1}$ component in the peripheral 2PCs arises largely from dijets: while the near-side jet peak is rejected by the $|\Delta \eta |~>~2$ requirement, the “away-side jet” correlation that arises from back-to-back jets and contributes at $\Delta \phi =\pi$, cannot be rejected entirely as its position varies in $|\Delta \eta |$ from event to event. In the peripheral multiplicity intervals, the away-side jet significantly affects the 2PC. It produces a large negative $v_{1,1}$ and also affects the other harmonics by adding alternately positive and negative contributions to $v_{n,n}$ harmonics of even and odd order, respectively. In peripheral events the $v_{n,n}$ are strongly biased by dijets especially at higher $p_{\mathrm{T}}$. The presence of the jets also results in the breakdown of the factorization relation (Eq. (3)).

### Scalar product and event plane analysis

The SP method was introduced by the STAR Collaboration [25] and is further discussed in Ref. [17]. The SP method is very similar to the Event Plane method (EP) widely used in earlier analyses [12, 13]. It is superior to the EP as $v_{n}\{\mathrm {SP}\}$ is an estimator of $\sqrt{\langle v_{n} ^{2}\rangle }$, independent of the detector resolution and acceptance, whereas $v_{n}\{\mathrm {EP}\}$ produces a detector-dependent estimate of $v_{n}$ that lies between $\langle v_{n} \rangle$ and $\sqrt{\langle v_{n} ^{2}\rangle }$ [28].

Both the SP and EP method use flow vectors $Q_{n}$ and $q_{n,j}$ defined as:

5 $$\begin{aligned} Q_{n} = |Q_{n}|\text {e}^{\text {i}n\Psi _{n}} = \frac{1}{M}\sum _{j=1,M}q_{n,j} = \frac{1}{M}\sum _{j=1,M}w_j \text {e}^{\text {i}n\phi _{j}}, \end{aligned}$$

where the sum runs over *M* particles in a single event. The $\phi _{j}$ is the particle azimuthal angle and *n* is the harmonic order. In this analysis the flow vectors are established separately for the two sides of the FCal and are denoted $Q_n^{N|P}$, where the N and P correspond to $\eta < 0$ and $\eta >0$ sides, respectively. The sum in Eq. (5) in this case runs over the calorimeter towers of approximate granularity $\Delta \eta \times \Delta \phi = 0.1 \times 0.1$ and the weights $w_i$ are the transverse energies $E_{\mathrm{T}}$ measured in the FCal towers. The flow vectors are also calculated using charged-particle tracks. In this case the sum in Eq. (5) is over tracks and $w_{j}$ is obtained as the MC tracking weight ($(1-f)/\epsilon$) multiplied by a factor that depends on azimuthal angle to correct for non-uniformity in the azimuthal-angle distribution of reconstructed tracks. This latter factor is obtained run-by-run from the data as the average track multiplicity in one $\eta$ slice of 0.1 divided by the multiplicity in the narrow $\Delta \eta \times \Delta \phi = 0.1 \times 0.1$ interval.

**Fig. 4:**
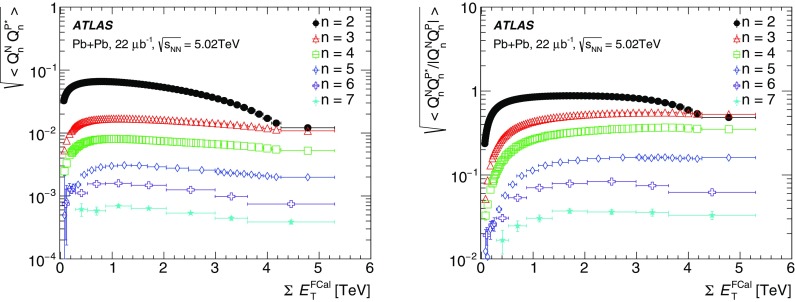
The dependence of the correction factor in the SP method, $\sqrt{\langle Q_{n}^{N}Q_{n}^{P*}\rangle }$ (left panel), and EP method, $\sqrt{\Big \langle \frac{Q_{n}^{N} Q_{n}^{P*}}{|Q_{n}^{N} ||Q_{n}^{P}|}\Big \rangle }$ (right panel), for all measured harmonics as a function of $\Sigma E_\mathrm {T}^\mathrm {FCal}$ binned according to the centrality bins definition

The main idea of the SP method is to correlate single-track unit flow vectors with the flow vector of all particles measured in the FCal region ($$3.2< |\eta | < 4.9$$). Therefore, the SP method differs from the two-particle correlation method, in which each single track is correlated with all tracks of $$|\Delta \eta |>2$$ in the event. The values of $$v_{n} $$ in this analysis are obtained as:

6 $$\begin{aligned} v_{n}\{\mathrm {SP}\}= & {} Re \frac{\langle q_{n,j}Q_{n}^{N|P*} \rangle }{\sqrt{\langle Q_{n}^{N}Q_{n}^{P*}\rangle }}\nonumber \\= & {} \frac{\langle |q_{n,j}||Q_{n}^{N|P}|\cos [n(\phi _{j} - \Psi _{n}^{N|P})] \rangle }{\sqrt{\langle |Q_{n}^{N}||Q_{n}^{P}|\cos [n(\Psi _{n}^{N} - \Psi _{n}^{P})] \rangle }}, \end{aligned}$$

where $q_{n,j}$ is the flow vector obtained for a small ($\eta , p_{\mathrm{T}}$) interval (typically 0.1 in $\eta$ and 0.1 $\mathrm{GeV}$ in $p_{\mathrm{T}}$below 5 $\mathrm{GeV}$ and 1 $\mathrm{GeV}$ at higher $p_{\mathrm{T}}$) using tracks, $Q_{n}^{N|P}$ is the flow vector obtained using either the N or P side of the FCal, chosen so that the $\eta$ gap between the $q_{n,j}$ and $Q_{n}$ is maximized, the * denotes complex conjugation, the $\Psi _{n}$ are estimates of the $n^{\text {th}}$-order reaction-plane angles (Eq. (1)) and the angular brackets indicate an average over all events. In the last line of Eq. (6) it is assumed that the sine terms disappear, as required from symmetry. The correction factor, $1/\sqrt{\langle Q_{n}^{N}Q_{n}^{P*}\rangle }$, (Eq. (6)) depends on the harmonic order and $\Sigma E_\mathrm {T}^\mathrm {FCal}$ as shown in the left panel of Fig. 4. The event-plane angles, $\Psi _{n}$, and the $Q_{n}$ vectors, both measured in the FCal, may be biased due to non-uniform detector response. As $\Psi _{n}$ varies randomly from event to event, its distribution should be uniform, and the components of the $Q_n$ vector, $Q_{n,x} = |Q_{n,}|\mathrm {cos}(\Psi _{n})$ and $Q_{n,y} = |Q_{n}|\mathrm {sin}(\Psi _{n})$, should be zero when averaged over many events. This is achieved by the following procedure. In its first step, non-zero offsets of the mean of raw flow vector coordinates are removed for each run: $Q_{n,i} =Q^{\mathrm {raw}}_{n,i} - \langle Q^{\mathrm {raw}}_{n,i}\rangle$ where $i=x,y$ and $\langle Q^{\mathrm {raw}}_{n}\rangle$ is the mean calculated for each run. However, even after this correction, residual higher-order non-uniformities persist, indicated by non-zero values of $\langle Q_{n,x}Q_{n,y}\rangle$. These are removed by rotating the $Q_{n}$ vector so that the corrected $Q_{n}$ vector has no skew ($\langle Q_{n,x}^{2}\rangle = \langle Q_{n,y}^{2}\rangle$; $\langle Q_{n,x} Q_{n,y}\rangle = 0$) and the distributions of the resulting EP angles, $\Psi _{n}$, are uniform [52].

In the Event Plane analysis the reference $Q_{n}$ vectors are normalized to unity, $Q_n^{N|P} \rightarrow Q_n^{N|P}/|Q_n^{N|P}|$, before using them in Eq. (6). So the $v_{n}$ estimate is obtained as:

7 $$\begin{aligned} v_{n} \{\mathrm {EP}\} = Re \frac{\Big \langle q_{n,j}\frac{Q_{n}^{N|P*}}{|Q_{n}^{N|P}|}\Big \rangle }{\sqrt{\Big \langle \frac{Q_{n}^{N}}{|Q_{n}^{N}|} \frac{Q_{n}^{P*}}{|Q_{n}^{P}|} \Big \rangle }} = \frac{\langle \cos [n(\phi _{j} - \Psi _{n}^{N|P})] \rangle }{\sqrt{\langle \cos [n(\Psi _{n}^{N} - \Psi _{n}^{P})] \rangle }}. \end{aligned}$$

The denominator of Eq. (7), shown in the right panel of Fig. 4, can be thought of as a resolution. It is distinct for each harmonic and depends on $\Sigma E_\mathrm {T}^\mathrm {FCal}$.

In this analysis the EP method is used only for the purpose of a direct comparison with the results obtained in Run 1 [13], in which only the EP method was used.

The analysis is performed in intervals of centrality. The $v_{n}$ values are obtained in narrow bins of $p_{\mathrm{T}}$ and $\eta$, which are summed, taking into account tracking efficiency and fake rate, to obtain the integrated results.

A detailed study based on a HIJING [39] Monte Carlo sample showed a difference for the most central events between the $v_{n}$ obtained with generated particles and the $v_{n}$ obtained with reconstructed tracks (the “MC closure test”). The discrepancies are due to the presence of fake tracks, which at low $p_{\mathrm{T}}$ distort the $v_{n}$ measurements, and a tracking inefficiency in the event-plane direction due to increased detector occupancy resulting from the flow phenomenon itself, which lowers the measured $v_{n}$ values. The study based on the $d_0$ distribution also showed that the fake-track rates are overestimated in MC simulation as compared to the data. This disagreement was removed by weighting MC tracks so that the $d_0$-distribution tails ($2< |d_0| < 10 \, \hbox {mm}$) match those in data, following the procedure described in Ref. [53]. It was observed that the contribution of fakes to the “MC non-closure” is significant only for events with centrality < 30% and at low $p_{\mathrm{T}}$, which is the region where the fake rate is the largest. In this modified MC sample, the relative differences between values of the $v_{n}$ measured with generated particles and reconstructed tracks are used as corrections to account for both effects; the fakes and the $\Psi _{n}$-dependent inefficiency. Corrections are at most 5–10%. For example, for $v_{2}$ in the 0–5% centrality interval, the correction is as large as 7% at low $p_{\mathrm{T}}$and becomes negligible above $p_{\mathrm{T}}> 2~\mathrm{GeV}$. Corrections of a similar magnitude are obtained for higher-order harmonics.

## Systematic uncertainties

The systematic uncertainties of the measured $v_{n}$ are evaluated by varying several aspects of the analysis. As the EP and SP results are subject to the same uncertainty sources, the uncertainty values are of the similar magnitude and are not discussed separately. Similarly, some of the uncertainty sources are common to the SP/EP and the 2PC methods and are discussed together. The uncertainties for two representative $p_{\mathrm{T}}$ intervals are summarized in the Tables 3 and 4 for the 2PC and SP/EP methods, respectively. In the discussion below, other $p_{\mathrm{T}}$ ranges are referred to if uncertainties are significantly higher than in the $p_{\mathrm{T}}$ ranges shown in the tables. The following sources of uncertainty are considered:

- **Track selection:** The tracking selection requirements control the relative contribution of genuine charged particles and fake tracks entering the analysis. The stability of the results to the track selection is evaluated by varying the requirements imposed on the reconstructed tracks. Two sets of variations are used. In the first case the required number of pixel and SCT hits on the reconstructed track are relaxed to one and six, respectively. Additionally, the requirements on the transverse and longitudinal impact parameters of the track are relaxed to 1.5 mm. In the second case, the track selection is based on requirements used for the baseline measurement, but the transverse and longitudinal impact parameters of the track are restricted to 0.5 mm. For each variation, the entire analysis is repeated including the evaluation of the corresponding efficiencies and fake rates. The fake rate is largest at the lowest $p_{\mathrm{T}}$(0.5 GeV) and for the most central events, and consequently the variation in the $v_{n}$ values obtained from this procedure is largest, typically 10%, in this region of phase space.
- **Tracking efficiency:** As mentioned above, the tracks are weighted by a factor $(1-f)/\epsilon (p_{\mathrm{T}},\eta )$ when calculating the $v_{n}$ to account for the effects of the tracking efficiency. Uncertainties in the efficiency, resulting e.g. from an uncertainty in the amount of detector material, need to be propagated into the measured $v_{n}$ [54]. This uncertainty is evaluated by varying the efficiency up and down within its uncertainties in a $p_{\mathrm{T}}$-dependent manner and re-evaluating the $v_{n}$. This contribution to the overall uncertainty is very small and amounts to less than 1% on average. This is because the change of efficiency largely cancels out in the differential $v_{n} (p_{\mathrm{T}})$ measurement, and for $v_{n}$ integrated over $p_{\mathrm{T}}$the low-$p_{\mathrm{T}}$particles dominate the measurement. It does not change significantly with centrality nor with the order of harmonics.
- **Centrality determination:** An uncertainty in the flow harmonics comes from the uncertainty in the fraction of the total inelastic cross-section accepted by the trigger and the event selection criteria, which was estimated to be at a level of 1%. The $v_{n}$ uncertainty is evaluated by repeating the analysis with the modified centrality selections on the $\Sigma E_\mathrm {T}^\mathrm {FCal}$ distribution shown in Fig. 1, that give the ± 1% uncertainty in the sampled fraction of the cross-section [12]. The changes in the $v_{n}$ are largest in the peripheral-centrality intervals, for which the bin definitions are significantly changed when remapping the centralities. For $v_{2}$, a change of $\sim$0.8% (2PC) and $\sim$1% (SP) is also observed in the most central events. This is because the $v_{2}$ changes rapidly with centrality in central events, so slight variations in the centrality definition result in significant change in $v_{2}$. For $v_{3}$ this uncertainty varies from less than 0.5% over the 0–50% centrality range to $\sim$5% in the 70–80% centrality interval. For the higher-order harmonics, $n>3$, the uncertainty is less than 0.5% over the 0–50% centrality range and increases to about 2% for more peripheral bins. The variation in the $v_{n}$ when using these alternative centrality definitions is taken as a systematic uncertainty. To limit the statistical instability of $v_{6}$ and $v_{7}$ in uncertainty estimation, the variations for this measurement were determined over a wide range of $p_{\mathrm{T}}$= 0.5–60 $\mathrm{GeV}$.
- **MC corrections:** To assess the uncertainty related to the MC corrections the closure test is repeated with the two selections of tracks described in the “track selection” paragraph. Differences between the correction factors obtained with loose, nominal and tight tracking selections are compared. The difference between them is largest at low $p_{\mathrm{T}}$and central events and amounts typically to a few percent. It is negligibly small above 2 $\mathrm{GeV}$. The larger of the two differences (between the nominal and loose tracking selections) is used as an uncertainty estimate.
- **Residual sine term:** The ability of the detector to measure small $$v_{n}$$ signals can be quantified by comparing the value of the $$v_{n}$$ calculated as the real part of the flow vector product (SP) in Eq. (6) with its imaginary part. The ratio $$Im(SP)/v_{n} $$ is taken as a contribution to the systematic uncertainty. The contribution from this source is $$\sim $$1% in most of the phase space, while for the higher harmonics ($$n=6, 7$$) it can reach 20% in the most central collisions. This uncertainty is only relevant for the $$v_{n}$$ values measured by the EP and SP methods.
- **Variation of FCal acceptance in the** $Q_n^{N|P}$ **estimation:** In order to quantify an uncertainty arising from the FCal acceptance in the $Q_n^{N|P}$ estimation, $v_{n}$ harmonics are compared for two distinct FCal regions $3.2<|\eta |<4$ and $4<|\eta |<4.8$ used for the determination of the reference flow vector, $Q_n$. The differences between the $v_{n}$ values are treated as the systematic uncertainty, which, similarly to the $\eta$ symmetry (next paragraph), quantifies the ability of the detector to measure small signals. Accordingly, this contribution is small ($\sim$ 1%) for $v_{2}$ and $v_{3}$ and starts growing for higher-order harmonics, reaching about 27% for $v_{7}$. This uncertainty is only relevant to the $v_{n}$ values measured by the EP and SP methods.
- **Detector non-uniformity:** Due to the symmetry of the Pb+Pb collision system the $v_{n} (\eta )$ are expected to be on average symmetric in rapidity. Any difference between the event-averaged $v_{n}$ at $\pm \eta$ arises from residual detector non-uniformity. The difference between the $v_{n}$ values measured in opposite hemispheres is treated as the systematic uncertainty quantifying non-perfect detector performance. This uncertainty is in general very low (less than 1%) except for high-order harmonics: $v_{5}$ and $v_{6}$ at high $p_{\mathrm{T}}$and $v_{7}$ at all $p_{\mathrm{T}}$. This uncertainty only contributes to the $v_{n}$ values measured by the EP and SP methods. For the 2PC method, the residual non-uniformity is estimated by varying the event-mixing procedure.
- **Event-mixing:** As explained in Sect. 4.1, the 2PC analysis uses the event-mixing technique to estimate and correct for the detector-acceptance effects. Potential systematic uncertainties in the $v_{n}$ due to the residual pair-acceptance effects, which were not removed by the mixed events, are evaluated by varying the multiplicity and *z*-vertex matching criteria used to make the mixed-event distributions, following Ref. [13]. The resulting uncertainty for $v_{2}$–$v_{5}$ is between 1–3%, and for $v_{6}$ is between 4–8% for most of the centrality and $p_{\mathrm{T}}$ ranges measured in this paper. However, the uncertainties for $v_{4}$–$v_{6}$ are significantly larger for $p_{\mathrm{T}}\ {<} ~0.7~\mathrm{GeV}$, where the $v_{n}$ signals are quite small and very susceptible to acceptance effects, and for $v_{6}$ are correlated with statistical uncertainties. The uncertainties are also significantly larger for $p_{\mathrm{T}} {>} ~10~\mathrm{GeV}$, where they are difficult to determine due to large statistical uncertainties in the measurements.

**Table 3:** The systematic uncertainties associated with the 2PC $v_{n}$ measurements for selected intervals of $p_{\mathrm{T}}$and for 5–10% and 40–50% centrality bins. The contributions are expressed in %. The total systematic uncertainty is obtained by adding the contribution of the individual sources in quadrature

Systematic sources	*n*th harmonic	5–10%		40–50%	
		0.8–1.0 $\mathrm{GeV}$	6–8 $\mathrm{GeV}$	0.8–1.0 $\mathrm{GeV}$	6–8 $\mathrm{GeV}$
Track selection	$v_{2}$	0.5	0.5	0.5	0.5
	$v_{3}$	1	1	0.5	0.5
	$v_{4}$	0.5	0.5	0.5	1
	$v_{5}$	2	0.5	0.5	5
	$v_{6}$	2	2	2	2
Tracking efficiency	$v_{2}$	0.1	0.1	0.1	0.1
	$v_{3}$	0.1	0.1	0.1	0.1
	$v_{4}$	0.1	0.1	0.1	0.1
	$v_{5}$	0.1	0.1	0.1	0.3
	$v_{6}$	1	0.1	1	0.1
Centrality determination	$v_{2}$	1	1	0.5	0.5
	$v_{3}$	0.5	0.5	0.5	3
	$v_{4}$	0.5	0.5	0.5	3
	$v_{5}$	0.5	0.5	0.5	3
	$v_{6}$	0.5	0.5	0.5	3
MC corrections	$v_{2}$	2	0.5	0.5	0.5
	$v_{3}$	2	0.5	0.5	0.5
	$v_{4}$	1	0.5	0.5	0.5
	$v_{5}$	1	0.5	1	1
	$v_{6}$	3	0.5	2	0.5
Event-mixing	$v_{2}$	1	1	1	1
	$v_{3}$	1	3	1	3
	$v_{4}$	2	6	1	6
	$v_{5}$	3	10	3	10
	$v_{6}$	5	15	5	15

**Table 4:** The systematic uncertainties associated with the SP and EP (in parentheses) $v_{n}$ measurements for $v_{n}$ in 5–10% and 40–50% centrality bins. The contributions are expressed in %. The total systematic uncertainty is obtained by adding the contribution of the individual sources in quadrature

Systematic sources	*n*th harmonic	5–10%		40–50%	
		0.8–1 $\mathrm{GeV}$	9–10 $\mathrm{GeV}$	0.8–1 $\mathrm{GeV}$	9–10 $\mathrm{GeV}$
Track selection	$v_{2}$	0.5 (1)	0.5 ($<0.5$)	$<0.5$ ($<0.5$)	$<0.5$ ($<0.5$)
	$v_{3}$	1 (1)	1 ($<0.5$)	0.5 (0.5)	0.5 (0.5)
	$v_{4}$	0.5 (0.5)	$<0.5$ (0.5)	$<0.5$ ($<0.5$)	1 (1)
	$v_{5}$	2 (1)	0.5 ($<0.5$)	0.6 (0.5)	5 (4)
	$v_{6}$	2 (2)		2 (2)	
	$v_{7}$	6 (6)		4 (5)	
Tracking efficiency	$v_{2}$	0.1 (0.1)		0.1 (0.1)	
	$v_{3}$	0.1 (0.1)		0.1 (0.1)	
	$v_{4}$	0.1 (0.1)		0.1 (0.1)	
	$v_{5}$	0.1 (0.1)		0.1 (0.1)	
	$v_{6}$	1 (1)	0.1 (0.1)	1 (1)	0.1 (0.1)
	$v_{7}$	1.5 (1.5)		1.5 (1.5)	
Centrality determination	$v_{2}$	0.5 (0.5)	0.5 (0.5)	$<0.5$ ($<0.5$)	$<0.5$ ($<0.5$)
	$v_{3}$	$<0.5$ ($<0.5$)	$<0.5$ ($<0.5$)	$<0.5$ ($<0.5$)	0.5 (1)
	$v_{4}$	$<0.5$ ($<0.5$)	$<0.5$ ($<0.5$)	0.5 (0.5)	$<0.5$ ($<0.5$)
	$v_{5}$	$<0.5$ ($<0.5$)	$<0.5$ (0.5)	1 (1)	1 (1)
	$v_{6}$	2 (2)		2 (3)	2 (3)
	$v_{7}$	2 (3)		5 (5)	
Residual sine term	$v_{2}$	0.5 (0.5)	0.5 (0.5)	0.5 (0.5)	0.5 (0.5)
	$v_{3}$	1 (1)	1 (1)	0.5 (1)	0.5 (0.5)
	$v_{4}$	1 (0.5)	1 (1)	1 (0.5)	1 (1)
	$v_{5}$	1 (0.5)	1 (1)	1 (1)	0.5 (0.5)
	$v_{6}$	22 (26)	2 (1)	19 (11)	1 (3)
	$v_{7}$	20 (20)		17 (4)	
MC corrections	$v_{2}$	2 (2)	$<0.5$ ($<0.5$)	$<0.5$ ($<0.5$)	$<0.5$ ($<0.5$)
	$v_{3}$	2 (2)	$<0.5$ ($<0.5$)	$<0.5$ ($<0.5$)	$<0.5$ ($<0.5$)
	$v_{4}$	1 (1)	$<0.5$ ($<0.5$)	$<0.5$ ($<0.5$)	$<0.5$ ($<0.5$)
	$v_{5}$	1 (1)	$<0.5$ (0.5 )	1 (1)	1 (0.5)
	$v_{6}$	3 (3)	$<0.5$ (0.5)	2 (2)	0.5 (0.5)
	$v_{7}$	–	–	–	–
FCal response	$v_{2}$	$<0.5$ (1)	0.5 (1)	$<0.5$ (0.5)	1 (1)
	$v_{3}$	0.5 (0.5)	0.5 (1)	$<0.5$ ($<0.5$)	2 (3)
	$v_{4}$	1 (2)	$<0.5$ ($<0.5$)	1 (1)	2 (2)
	$v_{5}$	1 (1)	3 (1)	4 (8)	9 (16)
	$v_{6}$	3 (5)		16 (14)	
	$v_{7}$	27 (34)		20 (9)	
Detector non-uniformity	$v_{2}$	$<0.5$ ($<0.5$)	$<0.5$ ($<0.5$)	$<0.5$ ($<0.5$)	$<0.5$ ($<0.5$)
	$v_{3}$	0.5 ($<0.5$)	$<0.5$ ($<0.5$)	$<0.5$ ($<0.5$)	$<0.5$ ($<0.5$)
	$v_{4}$	$<0.5$ (1)	$<0.5$ (0.5)	0.5 (0.5)	$<0.5$ (0.5)
	$v_{5}$	2 (2)	1 (0.5)	1 (0.5)	1 (0.5)
	$v_{6}$	8 (10)		0.5 (2)	
	$v_{7}$	2 (3)		18 (14)	

## Results

**Fig. 5:**
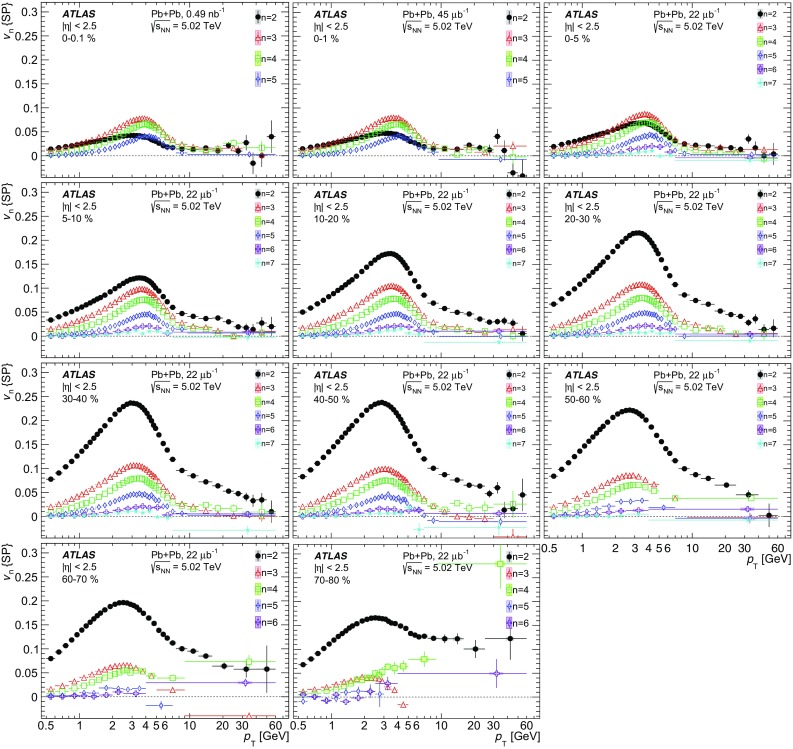
The $v_{n}$ obtained with the SP method as a function of transverse momentum $p_{\mathrm{T}}$, integrated over $|\eta | < 2.5$ in 11 centrality intervals, from the most central at the top left panel to the most peripheral at the bottom right panel. Results are averaged over the intervals indicated by horizontal error bars. The vertical error bars indicate statistical uncertainties; the shaded boxes indicate systematic uncertainties

**Fig. 6:**
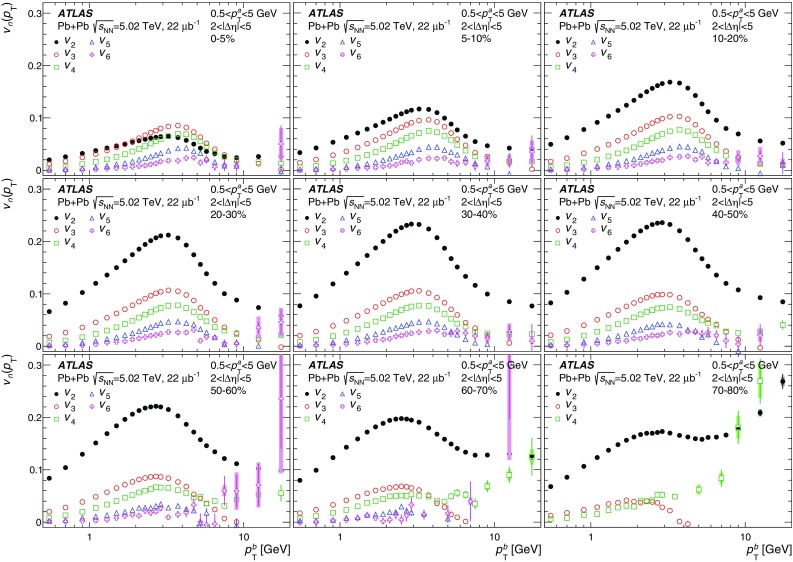
The $v_{n}$ values obtained with the 2PC method as a function of $p_{\mathrm {T}}^{b}$ for $0.5~<~p_{\mathrm {T}}^{a} <~5~\mathrm{GeV}$. Each panel represents a different centrality interval. The vertical error bars indicate statistical uncertainties. The shaded bands indicate systematic uncertainties

### The $p_{\mathrm{T}}$ dependence of $v_{n}$

Figures 5 and 6 show the $v_{n}$ obtained from the SP and 2PC methods, respectively, as a function of $p_{\mathrm{T}}$ for several centrality intervals. For the SP method the $v_{2}$–$v_{5}$ harmonics are also shown for the 0–0.1% and 0–1% ultra-central collisions. The SP results are integrated over the pseudorapidity $|\eta |<2.5$ and the 2PC results are obtained with $0.5~<~p_{\mathrm {T}}^{a} <~5~\mathrm{GeV}$ and for $2~<~|\Delta \eta |~<~5$. The $v_{n}$ values show a similar $p_{\mathrm{T}}$dependence across all centralities: a nearly linear rise to about 2 $\mathrm{GeV}$, followed by a gradual increase to reach a maximum around 2–4 $\mathrm{GeV}$ and a gradual fall at higher $p_{\mathrm{T}}$. However, significant $v_{n}$ values persist at high $p_{\mathrm{T}}$($\sim$20 $\mathrm{GeV}$). The $v_{2}$ is positive even at the highest measured $p_{\mathrm{T}}$of 60 $\mathrm{GeV}$ (Fig. 5). This indicates the parton energy loss in the created medium [30]. Such elliptic flow is expected due to path-length dependence of the energy loss of high-$p_{\mathrm{T}}$ partons traversing the hot and dense medium. In peripheral events, at the highest $p_{\mathrm{T}}$, the 2PC and SP $v_{2}$ values again show an increasing trend due to the increasing influence of the away-side jet. The increased $v_{2}$ in peripheral collisions at high-$p_{\mathrm{T}}$ is accompanied by reduced values of $v_{3}$ and increased values of $v_{4}$, which is characteristic of a large away-side peak, as described in Sect. 4.1. This is most clearly seen in the 70–80% centrality interval.

The $v_{2}$ varies significantly with centrality, reflecting a change in the shape of the average initial collision geometry, from nearly circular in ultra-central collisions to an almond shape in peripheral events. The higher harmonics do not show similar behaviour, as neither higher-order eccentricities nor the fluctuations vary so significantly with the centrality. The $v_{2}$ is dominant at all centralities, except for the most central collisions interval where, at intermediate $p_{\mathrm{T}}$, $v_{3}$ and $v_{4}$ become larger than $v_{2}$, indicating that the dominant source of observed flow comes from the initial geometry fluctuations. This change in the $v_{n}$ ordering is even more pronounced in the 1% and 0.1% ultra-central collisions measured using the SP method, which shows that, in the $p_{\mathrm{T}}$region around the $v_{n}$ peak, $v_{3}> v_{4} > v_{2} \approx v_{5}$. The $v_{4}$, similarly to $v_{2}$, exhibits an increase beyond $p_{\mathrm{T}}\sim 10\ \mathrm{GeV}$, which can be attributed to the presence of the events with dijets in the data. In the SP measurement the $v_{7}$ results are also presented. The characteristics of $v_{7}$ are similar to the other high-order harmonics, but the values are smaller and significant, given the uncertainties, only in central and mid-central collisions and for the $p_{\mathrm{T}}$ range of 2–6 $\mathrm{GeV}$.

#### The scalar product and event plane methods comparison

**Fig. 7:**
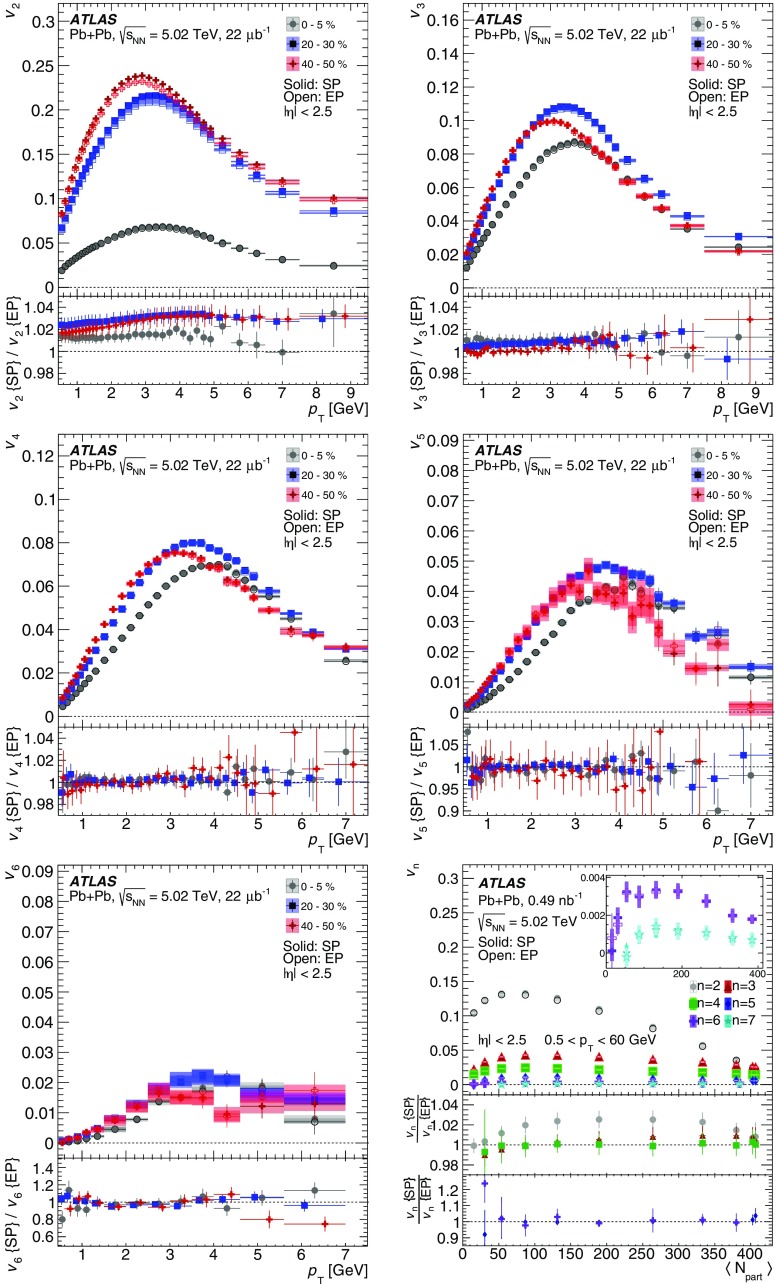
Comparison of the $v_{n}$ obtained with EP and SP methods as a function of $p_{\mathrm{T}}$ in three centrality bins: 0–5%, 20–30% and 40–50%. The right bottom panel shows the $v_{n}$ as a function of $N_{\mathrm {part}}$, integrated over $0.5~<~p_{\mathrm{T}}~<~60~\mathrm{GeV}$. The correspondence of $N_{\mathrm {part}}$ to centrality intervals is provided in Table 2. In the inset the $v_{6}$ and $v_{7}$ integrated over $0.5~<~p_{\mathrm{T}}~<~60~\mathrm{GeV}$ are shown with adjusted scale. For the $v_{n} (p_{\mathrm{T}})$ comparisons, the results are averaged over the intervals indicated by horizontal error bars. The vertical error bars indicate the quadrature sum of statistical and systematical uncertainties

Figure 7 compares the $v_{n}$ values measured with the EP and SP methods as a function of $p_{\mathrm{T}}$and $N_{\mathrm {part}}$ for the integrated $p_{\mathrm{T}}$ range of $0.5<p_{\mathrm{T}}<60~\mathrm{GeV}$. A small difference is seen between the $v_{2}$ values measured with the two methods. The difference is largest in mid-central events: about 3% in the 20–30% and 40–50% centrality intervals, about 1% in the 0–5% most central collisions and negligible in peripheral collisions. This difference is expected according to Ref. [28] as the SP method measures $\sqrt{\langle v_{n} ^2\rangle }$ while the EP method measures values between $\langle v_{n} \rangle$ and $\sqrt{\langle v_{n} ^2\rangle }$, with the former value attained in the limit of a small correction factor (the inverse of the denominator in Eq. (7)) and approaching the latter when the correction factor is large. In the most central and peripheral events, where the correction is large for the second-order harmonic, the EP $v_{2}$ values are closer to the SP ones, while for the mid-central events where the correction is small, the EP $v_{2}$ values are systematically lower than the SP $v_{2}$ values. Higher-order EP and SP $v_{n}$ harmonics are consistent with each other.

**Fig. 8:**
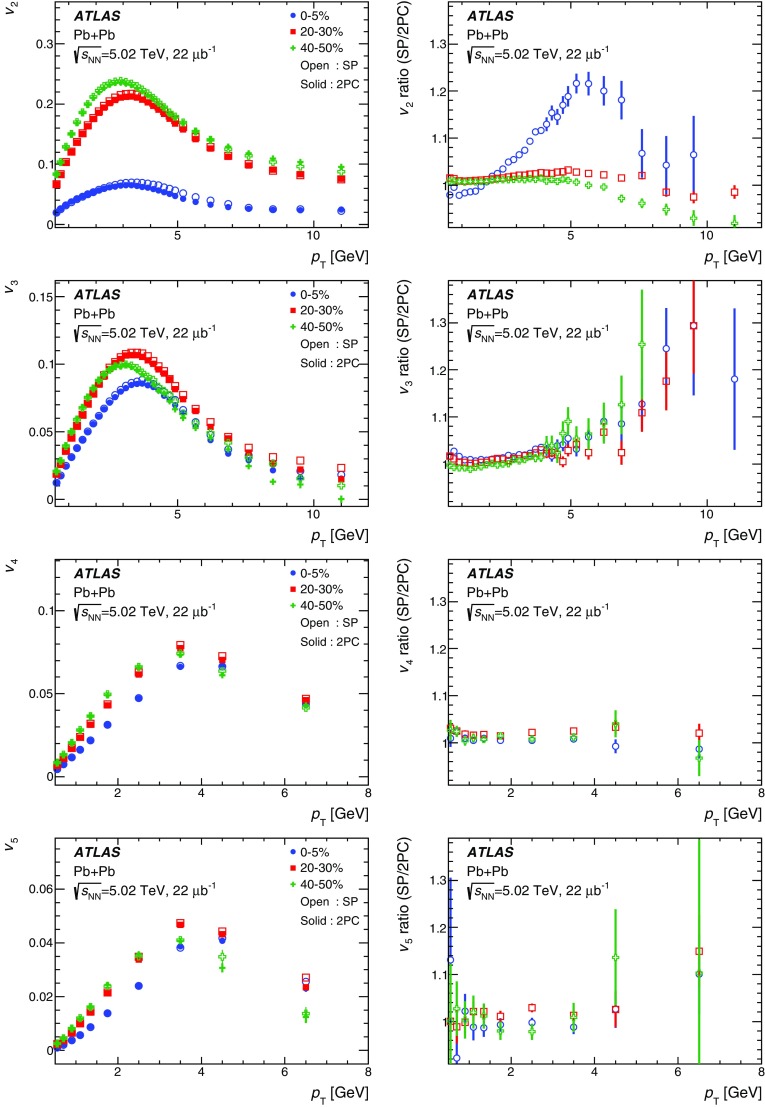
Comparison of the $v_{n}$ obtained with 2PC and SP methods as a function of $p_{\mathrm{T}}$. Each panel shows the comparison for a different order harmonic. The comparisons are shown for three different centrality intervals: 0–5%, 20–30% and 40–50%. The vertical error bars indicate statistical uncertainties only

#### The scalar product and two-particle correlation methods comparison

A comparison of the SP and 2PC results is presented in Fig. 8. In general, results from the two methods are quite consistent. There is a significant difference in $v_{2}$ from the two methods in the phase-space region $p_{\mathrm{T}}<5~\mathrm{GeV}$, 0–5% centrality. This difference decreases considerably for 20–30% mid-central events, where the $v_{2}$ values match within 2–5% up to $p_{\mathrm{T}}\sim$10 $\mathrm{GeV}$. For $v_{3}$–$v_{5}$, where there are enough events for a clear comparison, the $v_{n}$ values match within $\sim$4% for $p_{\mathrm{T}}<4~\mathrm{GeV}$ for the three centrality intervals shown in Fig. 8. In principle, both the SP and 2PC methods measure $\sqrt{\langle v_{n} ^2\rangle }$ and the flow harmonics measured by the two methods should be identical. However, a breakdown of factorization (Eq. (3)) results in systematic differences in the flow harmonics measurement. Such factorization breakdown has been observed to be significant for $v_{2}$ in central events [55], and in general for all $v_{n}$ at higher $p_{\mathrm{T}}$, and is the leading source of disagreement between the 2PC and SP results. Furthermore, in the 2PC method the $\Delta \eta$ gap between the reference and associated particles is chosen to be $|\Delta \eta |~>~2$, while in the SP method, where the reference flow is measured in the FCal, the minimum gap between the tracks and the FCal is 3.2 units in $\eta$. The presence of longitudinal-flow fluctuations, in which the event-plane angle can change with $\eta$, can result in different $v_{n}$ values depending on the $\eta$ range where the reference flow is measured [27, 56]. This effect is also found to be larger in central events and relatively smaller in mid-central events [56]. These effects can further contribute to the observed difference between the SP and 2PC $v_{n}$ values.

#### Comparison to Pb+Pb results at $\sqrt{s_{_\text {NN}}}$ = 2.76 $\mathrm{TeV}$

**Fig. 9:**
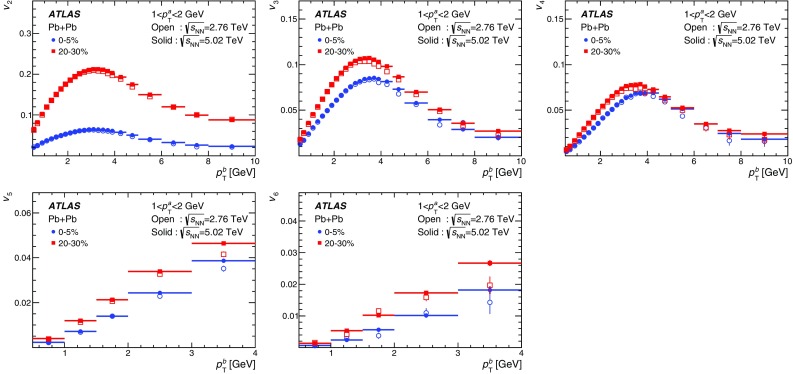
Comparisons of the 2PC $v_{n}$ harmonics measured at $\sqrt{s_{_\text {NN}}}$ = 2.76 $\mathrm{TeV}$ (Run 1) and at $\sqrt{s_{_\text {NN}}}$ = 5.02 $\mathrm{TeV}$ (Run 2). The results are plotted as a function of $p_{\mathrm {T}}^{b}$ for $1~<~p_{\mathrm {T}}^{a} ~<~2~\mathrm{GeV}$ for two centralities: 0–5% and 20–30%. Each panel corresponds to a different harmonic. Results are averaged over the intervals indicated by horizontal error bars. The vertical error bars indicate statistical uncertainties

**Fig. 10:**
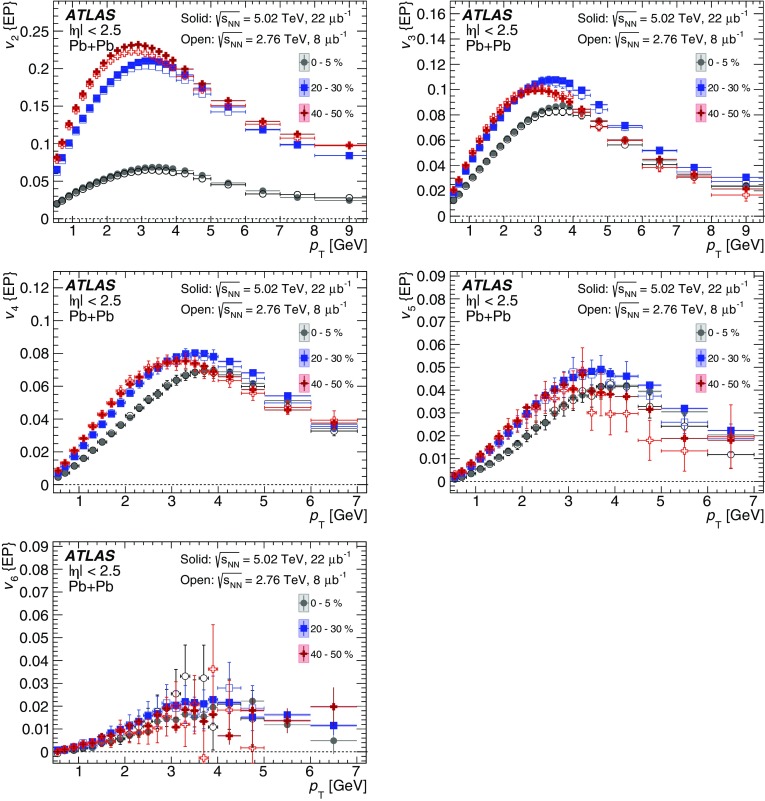
Comparison of the $v_{n}$ obtained with EP method using Run 1 and Run 2 data as a function of $p_{\mathrm{T}}$. The results are shown in three centrality intervals: 0–5%, 20–30% and 40–50%. Results are averaged over the intervals indicated by horizontal error bars. The vertical error bars indicate statistical uncertainties. The shaded areas indicate systematic uncertainties

Figure 9 shows a comparison of the $v_{n}$ measured in the present analysis at $\sqrt{s_{_\text {NN}}}=5.02$ $\mathrm{TeV}$ with the corresponding measurements at $\sqrt{s_{_\text {NN}}}=2.76$ $\mathrm{TeV}$ for harmonics $v_{2}$ to $v_{6}$ obtained using the 2PC method [13]. The comparisons are shown for two centralities: a central interval of 0–5% and a mid-central interval of 20–30%. Figure 10 shows a similar comparison of results obtained using the EP method for 0–5%, 20–30% and 40–50% centrality bins. The $v_{n}$ at the two energies are quite similar and almost consistent throughout within systematic and statistical uncertainties, even though the MC non-closure correction was applied only in the $\sqrt{s_{_\text {NN}}}=5.02$ $\mathrm{TeV}$ measurement. These results are consistent with the recent ALICE measurements comparing the measurement of $v_{n}$ at the two collision energies [29].

**Fig. 11:**
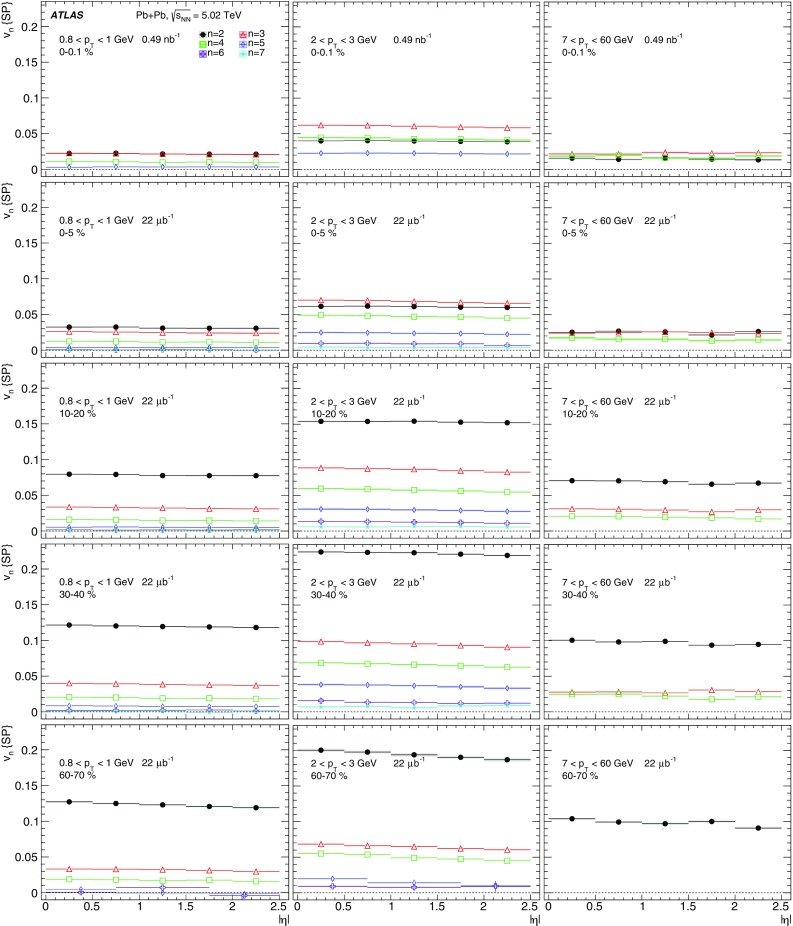
The $v_{n}$ as a function of pseudorapidity obtained with the SP method, for transverse momentum ranges: $0.8<p_{\mathrm{T}}<1~\mathrm{GeV}$ (left column), $2<p_{\mathrm{T}}<3~\mathrm{GeV}$ (middle column) and $7< p_{\mathrm{T}}< 60~\mathrm{GeV}$ (right column) and for centrality intervals: 0–0.1% (top row), 0–5%, 10–20%, 30–40% and 60–70% (bottom row). The vertical error bars indicate statistical uncertainties. The shaded boxes indicate systematic uncertainties

### The $\eta$ dependence of $v_{n}$

The pseudorapidity dependence of the $v_{2}$–$v_{7}$ obtained from the SP method is shown in Fig. 11 as a function of $|\eta |$. Benefiting from the symmetry of $v_{n} (\eta )$ with respect to $\eta =0$, the $v_{n}$ pseudorapidity dependence over the full range of pseudorapidity was folded into the $\eta$ range 0–2.5. The $\eta$-dependence is shown for three ranges of transverse momenta $0.8<p_{\mathrm{T}}<1~\mathrm{GeV}$, $2<p_{\mathrm{T}}<3~\mathrm{GeV}$ and $7<p_{\mathrm{T}}<60~\mathrm{GeV}$ and for five centrality intervals 0–0.1%, 0–5%, 10–20%, 30–40% and 60–70%. The strong dependence of flow harmonics on $p_{\mathrm{T}}$and centrality shown across different panels (all vertical axes in Fig. 11 have the same range) is discussed in the previous section. On the other hand, no strong pseudorapidity dependence of $v_{n}$ harmonics is observed. The $v_{2}$ harmonic in central and mid-central collisions for $p_{\mathrm{T}}<3~\mathrm{GeV}$ drops only by about 2–4% over the pseudorapidity range $|\eta |$ = 0–2.5. For peripheral collisions and for high $p_{\mathrm{T}}>7~\mathrm{GeV}$ a larger decrease of about 10% is observed. The $v_{3}$ harmonic in central and mid-central collisions over the $p_{\mathrm{T}}$ range from 2 to 3 $\mathrm{GeV}$ decreases by about 10% with a larger drop of $\sim$15% for peripheral collisions. Similar pseudorapidity dependence is measured for the $v_{4}$ harmonic in central and mid-central collisions over the $p_{\mathrm{T}}$ range from 2 to 3 $\mathrm{GeV}$ where it changes by about 10%, but a larger drop of 25% is observed in peripheral collisions. In other cases, $v_{n}$ harmonics are almost consistent with a uniform distribution within the statistical and systematic uncertainties. As observed in the earlier measurement of $v_{n}$ harmonics in 2.76 $\mathrm{TeV}$ Pb+Pb collisions [19], such a weak $\eta$ dependence of $v_{2}$ may be partially attributed to a contribution of “non-flow” short-range two-particle correlations.

### Centrality dependence of $v_{n}$

**Fig. 12:**
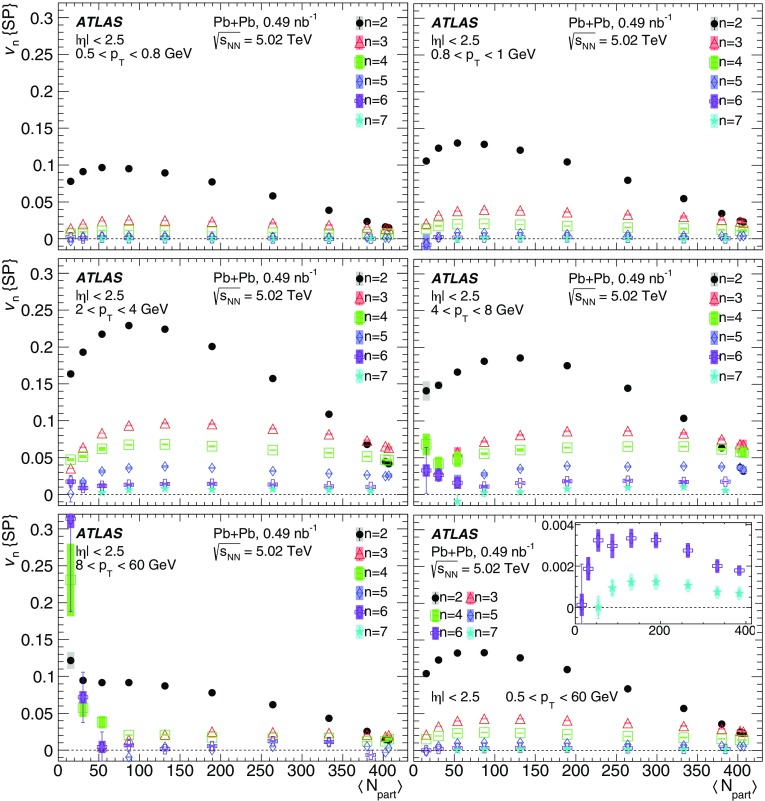
Integrated $v_{n}\{\mathrm {SP}\}$ vs. $N_{\mathrm {part}}$ for six $p_{\mathrm{T}}$ranges shown in the panels from lowest $p_{\mathrm{T}}$range at the top left to the highest at the bottom middle, measured using the scalar-product method. In the inset in the bottom-right panel the $v_{6}$ and $v_{7}$ integrated over $0.5<p_{\mathrm{T}}<60~\mathrm{GeV}$ are shown with adjusted scale. The correspondence of $N_{\mathrm {part}}$ to centrality intervals is provided in Table 2. Results are averaged over the intervals indicated by horizontal error bars. The vertical error bars indicate statistical uncertainties. The shaded areas indicate systematic uncertainties

Figure 12 shows the $N_{\mathrm {part}}$ dependence of $v_{n}$ integrated over $|\eta |<2.5$ and for various ranges of $p_{\mathrm{T}}$using the SP method. The elliptic flow is the dominant anisotropy, except at the largest $N_{\mathrm {part}}$ ($N_{\mathrm {part}}\gtrsim 350$), which corresponds to the 0-5% most central collisions. For $p_{\mathrm{T}}<8~\mathrm{GeV}$, a clear dependence on initial geometry can be observed as $v_{2}$ is highest in mid-central collisions, where this asymmetry is most significant. For $p_{\mathrm{T}}>8 ~\mathrm{GeV}$, $v_{2}$ is still the dominant harmonic, and it is non-zero even in peripheral collisions as non-flow effects start to contribute in this region. A hierarchy $v_{n+1} < v_n$ is observed for $n=2$–7 for all ranges of $p_{\mathrm{T}}$ and all centralities, except for the two bins 0–0.1% and 0–1% of the ultra-central collisions at intermediate $p_{\mathrm{T}}$, which are characterised by the flow harmonics ordering $v_{3}> v_{4} > v_{2} \approx v_{5}$.

### $v_{n} (p_{\mathrm{T}})$ scaling

**Fig. 13:**
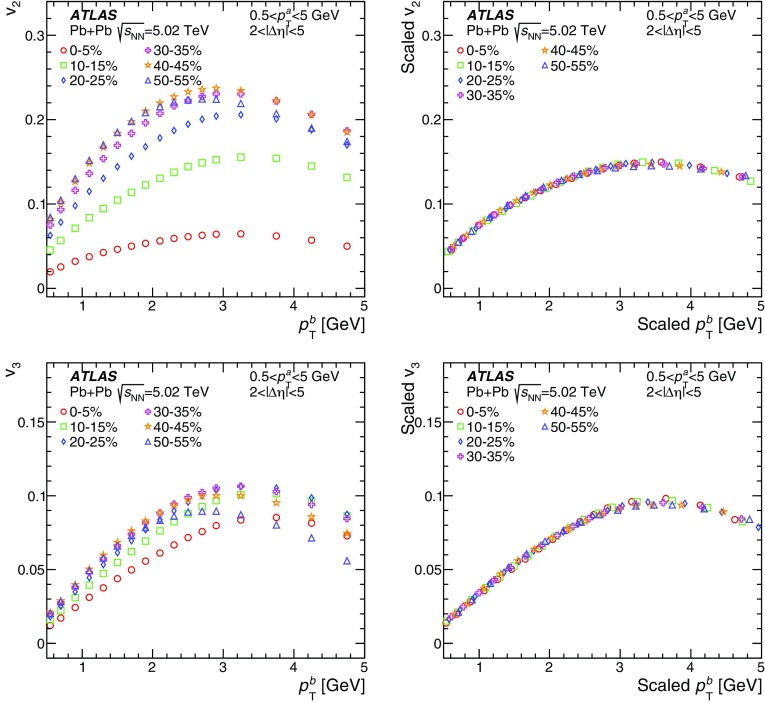
Left panels: the $v_{2}$ ($p_{\mathrm{T}}$) (top) and $v_{3}$ ($p_{\mathrm{T}}$) (bottom) for different centrality intervals. Right panels: the corresponding scaled-$v_{n}(p_{\mathrm{T}})$. The error bars indicate statistical and systematic uncertainties added in quadrature and are typically too small to be seen

The left panels of Fig. 13 compare the $p_{\mathrm{T}}$ dependence of $v_{2}$ and $v_{3}$ for different centrality intervals obtained from the 2PC method. Two distinct features are observed to change in the shape of the $v_{n} (p_{\mathrm{T}})$ (for the same *n*) between the different centrality intervals:

1. Change in the $$v_{n}$$-scale: The overall magnitude of the $$v_{n} $$ changes from one centrality to another. This effect is particularly large for $$v_{2}$$, and has to do with the changing of the average collision geometry from one centrality to another.
2. Change in the $$p_{\mathrm{T}}$$ scale: The $$p_{\mathrm{T}}$$ value at which the $$v_{n} $$ reaches its maximum also changes systematically from one centrality to another.

In a recent ATLAS paper [45] it was observed that for a given order *n*, the $$v_{n} (p_{\mathrm{T}})$$ in *p*+Pb and Pb+Pb collisions have a very similar $$p_{\mathrm{T}}$$ dependence. In fact, after a scaling along the $$p_{\mathrm{T}}$$ axis to account for the difference in $$\langle p_{\mathrm{T}}\rangle $$ between *p*+Pb and Pb+Pb collisions, and an empirical scaling along the $$v_{n} $$ (i.e. *y*-axis) to account for the difference in the collision geometry between the two systems, the scaled $$v_{n} (p_{\mathrm{T}})$$ in *p*+Pb and Pb+Pb collisions were found to be nearly identical. In this section, a check is done to see if such a scaling along the $$p_{\mathrm{T}}$$ and $$v_{n} $$ axes can yield universal shapes for the $$v_{n} $$ across the different centrality classes. Accordingly, the $$v_{n}(p_{\mathrm{T}})$$ are scaled along the *x*- and *y*-axes to match their shapes across the different centrality intervals. For the matching, the 0–60% centrality interval is chosen as the reference, and the $$v_{n} (p_{\mathrm{T}})$$ for the individual 5%-wide centralities are scaled to match best the $$v_{n} (p_{\mathrm{T}})$$ in the 0–60% centrality interval over the 0.5–5 $$\mathrm{GeV}$$ $$p_{\mathrm{T}}$$ range, with the scales along the *x*- and *y*-axes treated as fit parameters. The right panels of Fig. 13 show the scaled-$$v_{n}$$, for $$n=2$$ and 3. Overall, the $$v_{n} (p_{\mathrm{T}})$$ shapes match well after the $$p_{\mathrm{T}}$$ and $$v_{n} $$ scalings.

**Fig. 14:**
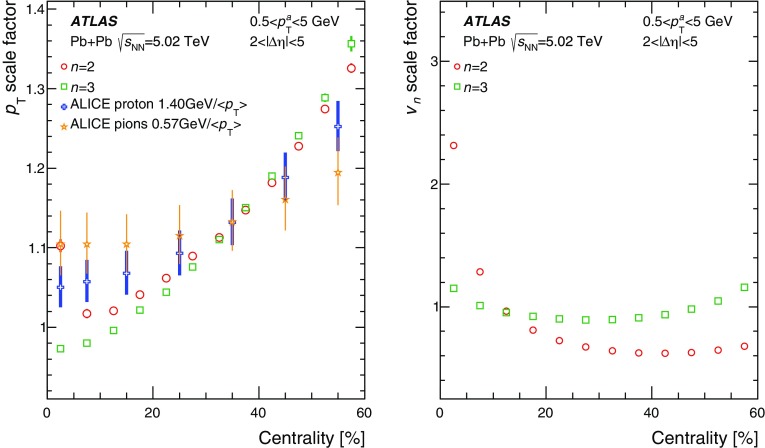
Left panel: the *x*-scale factors for the $v_{2}(p_{\mathrm{T}})$ and $v_{3}(p_{\mathrm{T}})$ (see text) as a function of the collision centrality. Also shown for comparison are the ALICE 1/$\langle p_{\mathrm{T}}\rangle$ for positively charged protons and pions (scaled by constant factors of 1.4 $\mathrm{GeV}$ and 0.57 $\mathrm{GeV}$, respectively for plotting purposes). Right panel: the *y*-scale factors. The error bars on the scale factors and on the ALICE data indicate systematic and statistical uncertainties added in quadrature

Figure 14 shows the *x* (or $p_{\mathrm{T}}$) and *y*-scale factors obtained for $v_{2}$ and $v_{3}$ as a function of centrality. It is interesting to note that the $p_{\mathrm{T}}$-scale factors are quite comparable between $v_{2}$ and $v_{3}$ across most of the centrality ranges. However, in the 0–10% most central events, some significant difference is observed between the two scale factors. This difference could be due to larger jet-bias and factorization-breaking effects in the $v_{2}$ as compared to $v_{3}$. The right panel of Fig. 14 shows the *y*-scale factors for $v_{2}$ and $v_{3}$ as well. Their centrality dependence is very different for the two harmonics. This is to be expected as the *y*-scale factors are mostly indicative of the changing collision geometry, which becomes more and more elliptic from central to mid-central events resulting in a large increase in $v_{2}$, while $v_{3}$, which is driven by fluctuations, changes only gradually.

In order to check if the change in $$\langle p_{\mathrm{T}}\rangle $$ between the different centralities accounts for the change in the *x*-scale of the $$v_{n} $$, the $$1/\langle p_{\mathrm{T}}\rangle $$ for protons and pions, as measured by the ALICE Collaboration [57], are also shown for comparison. While the centrality dependence of these $$1/\langle p_{\mathrm{T}}\rangle $$ factors is qualitatively similar to the scale factors for the $$v_{n} $$, the relative variation in $$1/\langle p_{\mathrm{T}}\rangle $$ is significantly smaller than the variation in the *x*-scale of the $$v_{n}$$, indicating that there are additional effects at play besides the change in the mean $$p_{\mathrm{T}}$$. However, whatever the origin of these effects may be, they result in nearly identical scaling factors for $$v_{2}$$ and $$v_{3}$$.

## Summary

This paper presents the first ATLAS measurements of azimuthal anisotropy of charged particles in Pb+Pb collisions at 5.02 $$\mathrm{TeV}$$ collected during LHC running in 2015. The measurements are based on the Pb+Pb sample of 0.49 $${\mathrm {nb}}^{-1}$$ integrated luminosity and are performed with the two-particle correlation, scalar-product and event-plane methods. The azimuthal anisotropy is quantified by the flow harmonics $$v_{2} $$–$$v_{6} $$ and $$v_{2} $$–$$v_{7} $$ for measurements based on the 2PC and SP/EP methods, respectively. The flow harmonics are obtained in wide transverse momentum ($$0.5< p_{\mathrm{T}}< ~ 60~\mathrm{GeV}$$), pseudorapidity ($$|\eta |< 2.5$$) and centrality 0–80% ranges. All harmonics show a similar trend in the $$p_{\mathrm{T}}$$dependence; first increasing with $$p_{\mathrm{T}}$$up to a maximum around 3–4 GeV and then decreasing for higher $$p_{\mathrm{T}}$$. Significant values of the second-order harmonic $$v_{2}$$ persist up to 60 $$\mathrm{GeV}$$. The $$v_{2}$$ results at high $$p_{\mathrm{T}}$$provide a useful handle for the study of partonic energy loss in the dense medium, and so can improve our understanding of the QGP properties. The values of the flow harmonics decrease strongly with increasing harmonic order for all centralities, except for 0–5% central collisions where a different ordering is seen: $$v_{3}> v_{4} > v_{2} \approx v_{5} $$ for $$p_{\mathrm{T}}$$above 1 $$\mathrm{GeV}$$, which is indicative of the presence of significant $$v_{2}$$ fluctuations in these ultra-central collisions. The elliptic flow signal is strongly dependent on event centrality and is largest in mid-central collisions of 30–50%. The higher-order harmonics show a weak centrality dependence, which is consistent with an anisotropy associated with fluctuations in the initial geometry. After scaling the $$p_{\mathrm{T}}$$and magnitude of the differential elliptic and triangular, $$v_{3}$$, flow harmonics at different centralities to best match the reference $$v_{2}$$ and $$v_{3}$$ for the 0–60% centrality interval over the $$p_{\mathrm{T}}$$range $$0.5~<p_{\mathrm{T}}<~5~\hbox {GeV}$$, the shapes of the rescaled harmonics agree well, indicating similarity among $$p_{\mathrm{T}}$$-shapes of flow harmonics evolving from different initial conditions. The $$v_{n}$$ coefficients are shown to exhibit a weak $$\eta $$-dependence, irrespective of the harmonic order, centrality and $$p_{\mathrm{T}}$$. A weaker pseudorapidity dependence of $$v_{n} (\eta )$$ in more central collisions is suggested by data. The results obtained using the EP and SP methods are consistent for harmonics of order $$n\ge 3$$. A small, systematic difference is observed for $$v_{2}$$, where the values obtained from the SP method are up to 3% larger than the values obtained using the EP method. The 2PC and SP methods give values for $$v_{n}$$ that are quite consistent up to $$\sim $$10 $$\mathrm{GeV}$$. However, in the most central events the SP method gives systematically larger values for $$v_{2}$$ for $$p_{\mathrm{T}}> 2~\mathrm{GeV}$$. Comparisons with measurements in Pb+Pb collisions at $$\sqrt{s_{_\text {NN}}}=2.76~\mathrm{TeV}$$ show that the $$p_{\mathrm{T}}$$dependence of the $$v_{n}$$ shows no change from $$\sqrt{s_{_\text {NN}}}=2.76~\mathrm{TeV}$$ to $$\sqrt{s_{_\text {NN}}}=5.02~\mathrm{TeV}$$.

The set of results on flow harmonics presented in this paper provides a tool for studies of the underlying mechanism leading to the large azimuthal anisotropy observed in the Pb+Pb collision system, and can be used to constrain the theoretical modelling of the initial state of the QGP, its subsequent hydrodynamic evolution as well as partonic energy loss in the dense and hot medium.

## Footnotes

1 ATLAS uses a right-handed coordinate system with its origin at the nominal interaction point (IP) in the centre of the detector and the *z*-axis along the beam pipe. The *x*-axis points from the IP to the centre of the LHC ring, and the *y*-axis points upward. Cylindrical coordinates $(r,\phi )$ are used in the transverse plane, $\phi$ being the azimuthal angle around the *z*-axis. The pseudorapidity is defined in terms of the polar angle $\theta$ as $\eta =-\ln \tan (\theta /2)$.
